# Gallium–Carbon: A Universal Composite for Sustainable 3D Printing of Integrated Sensor–Heater–Battery Systems in Wearable and Recyclable Electronics

**DOI:** 10.1021/acsami.4c02706

**Published:** 2024-06-15

**Authors:** Elahe Parvini, Abdollah Hajalilou, João Pedro Gonçalves Vilarinho, Pedro Alhais Lopes, Miguel Maranha, Mahmoud Tavakoli

**Affiliations:** Soft and Printed Microelectronics Lab, Institute of Systems and Robotics, University of Coimbra, Coimbra 3030-290, Portugal

**Keywords:** liquid metal composite, Ga–CB–SIS ink, 3D printable ink, sinter-free, recyclable, wearable thermal devices, strain sensor, energy storage device

## Abstract

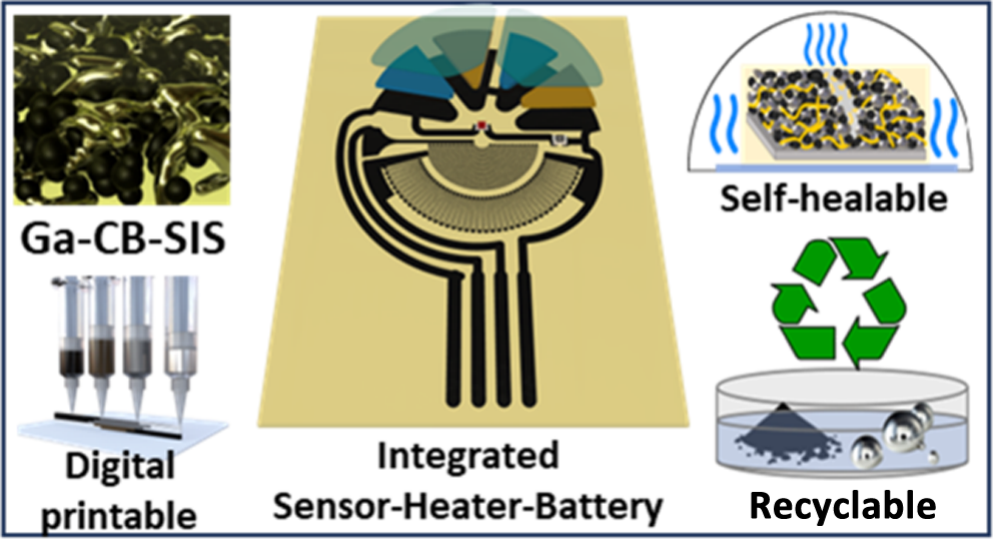

This study presents a novel three-dimensional (3D) printable gallium–carbon black–styrene isoprene styrene block copolymer (Ga–CB–SIS), offering a versatile solution for the rapid fabrication of stretchable and integrated sensor–heater–battery systems in wearable and recyclable electronics. The composite exhibits sinter-free characteristics, allowing for printing on various substrates, including heat-sensitive materials. Unlike traditional conductive inks, the Ga–CB–SIS composite, composed of gallium, carbon black, and styrene isoprene block copolymers, combines electrical conductivity, stretchability, and digital printability. By introducing carbon black as a filler material, the composite achieves promising electromechanical behavior, making it suitable for low-resistance heaters, batteries, and electrical interconnects. The fabrication process involves a simultaneous mixing and ball-milling technique, resulting in a homogeneous composition with a CB/Ga ratio of 4.3%. The Ga–CB–SIS composite showcases remarkable adaptability for digital printing on various substrates. Its self-healing property and efficient recycling technique using a deep eutectic solvent contribute to an environmentally conscious approach to electronic waste, with a high gallium recovery efficiency of ∼98%. The study’s innovation extends to applications, presenting a fully digitally printed stretchable Ga–CB–SIS battery integrated with strain sensors and heaters, representing a significant leap in LM-based composites. This multifunctional and sustainable Ga–CB–SIS composite emerges as a key player in the future of wearable electronics, offering integrated circuits with sensing, heating, and energy storage elements.

## Introduction

Soft, stretchable, and thin-film electronic circuits have a potentially transformative impact in many fields of research.^1,2^ This includes wearable technology and medical devices that are soft and can conform to the contours of the body.^3−5^ This requires stretchable substrates and conductors that ensure the devices are functional, flexible, and durable.^6−8^ Ga-based liquid metals (LMs) offer a unique combination of high electrical conductivity and fluidic deformability.^9−11^ This makes them ideal materials for use in flexible and stretchable circuits that should maintain their property despite suffering from multiple cycles of strain. Therefore, it has been desired to develop materials and methods for the digital printing of liquid Ga. However, the printing of gallium-based LMs is challenging due to their low viscosity, high surface tension, and smearing behavior.^12^ Maintaining the desired pattern and preventing unintended spreading or droplet formation can be challenging.^13^ To address them, researchers have created various types of liquid metal composites. One of the methods to create gallium composites involves dispersing liquid metal in elastomers, leading to the formation of novel composite materials.^14^ These materials show promise for a range of potential applications, including soft robotics,^15,16^ self-healing electronic devices,^17^ sensors, and intelligent heating devices.^18^ As an illustration, Wang et al. created a stretchable wearable electrically driven heater by employing Galinstan (GaInSn) liquid metal (LM) mixed with polydimethylsiloxane (PDMS) and printed using direct ink writing (DIW) on a silicon substrate. The design, featuring a micro-three-dimensional (micro-3D) conductive network and a sinusoidal structure, exhibited exceptional performance, specifically for thermotherapy during intense knee joint exercises.^19^ Furthermore, it demonstrated potential applications in stretchable capacitive strain sensors and stretchable earphones for wearable devices.^20−22^ Despite that, such composites, the elastomer–LM composite itself is nonconductive and requires mechanical sintering for conductivity.^23,24^ Although previous work identified limitations of LM elastomers (LMEs),^3,25−27^ solutions involve developing a free-sinter, nonsmearing, and distinctive conductive ink through the mixture of LM with metal filler particles and elastomers, effectively addressing the aforementioned issues.^3,24,28^ However, challenges include the excessive cost of filler particles such as Ag and Au, as well as their rigid behavior, posing practical application challenges.^3^ Previous approaches introduced rigid filler particles such as Ag, Au, Ni, Fe, etc., to achieve sinter-free conductivity (as indicated in Tables S1 and S2, SI). While these composites demonstrate good electromechanical (EM) properties at relatively high strains, challenges persist due to filler particle rigidity, high filler particle requirement for desired conductivity, cost and sustainability of particles, printability, and application versatility. For example, several works have focused on Ag–EGaIn–polymer composites. However, silver is costly, and both Ag and LM are difficult to recycle from the composite, due to the formation of binary intermetallic compounds like AgIn_2_.^29^ To further solve such issues, this study introduces a groundbreaking Ga–CB–SIS composite comprising gallium (Ga), carbon black (CB), and styrene isoprene block copolymers (SIS). Compared to previous works, this composite is lower in cost and more sustainable as it substitutes metals such as silver with carbon. Moreover, this composite is digitally printable and sinter-free. This eliminates the need for thermal sintering, which is time-consuming and energy-wasting. Thermal sintering is also an obstacle against multilayer 3D printing because every layer should be sintered individually. The composite has excellent adhesion to a wide range of substrates, thus permitting printing over several substrates.

Moreover, one particular advantage of this composite is that it can be used for various functionalities, including interconnects, sensing, heating, and most importantly as an electrode for energy storage. The composite features a self-healing property upon exposure to solvent vapor, providing an effective circuit repair mechanism. The combination of the above properties was not shown in any previous work.

In addition, the study adopts an environmentally conscious approach by demonstrating the recyclability of the composite and employing a deep eutectic solvent (DES) for gallium recovery with a high efficiency. Beyond material properties, the innovation extends to applications, presenting a fully digitally printed stretchable Ga–CB–SIS sensor–heater–battery system for wearable and recyclable electronics. This additive manufacturing (AM) approach represents a significant advancement in LM-based composites, integrating sensor, battery, and heating functionalities into a single digitally printed device. The selection of Ga–CB–SIS followed a comprehensive study of various carbon fillers, highlighting the meticulous optimization process aimed at achieving the desired combination of properties through different mixing protocols and compositions.

We optimized the composite to be digitally printed using a simple extrusion printer. This enables rapid prototyping of custom design circuits and personalized patches with the desired size of the battery, sensors, and interconnects. Using this composite, we demonstrate various circuits including a printed wearable glove for monitoring the hand gesture, a printed heater, and a printed battery, in which the Ga–CB–SIS is used as the anode electrode. Moreover, the composite is sinter-free and benefits from excellent adhesion properties, thanks to the inclusion of the SIS hyperelastic binder. These two properties permit printing Ga–CB–SIS over a wide range of substrates, including heat-sensitive substrates. This is important because it opens doors for printing over a wide range of substrates not previously possible, including plastics, textiles, paper, and a new generation of biodegradable and recyclable polymers that are often heat-sensitive. Some of these polymers melt at low temperatures and cannot withstand the typical sintering temperature of Ag-based inks (typically 150–300 °C).

Note that although gallium’s melting point is 29.76 °C Ga stays in the liquid phase for temperatures below its melting point. This is due to the supercooling effect of gallium, which was shown in previous works. In one work, it was found possible to supercool liquid gallium to −28 °C, or 58 °C below its melting point.^30^ In another work, it was found that the native gallium oxide enhances the supercooling as it prevents the liquid from contacting nucleating surfaces.^31^ However, the freezing of Ga was reported to be −15 ± 3.5 °C. Therefore, once melted, gallium can maintain its liquid phase for temperatures significantly below its melting point.

**Figure 1:**
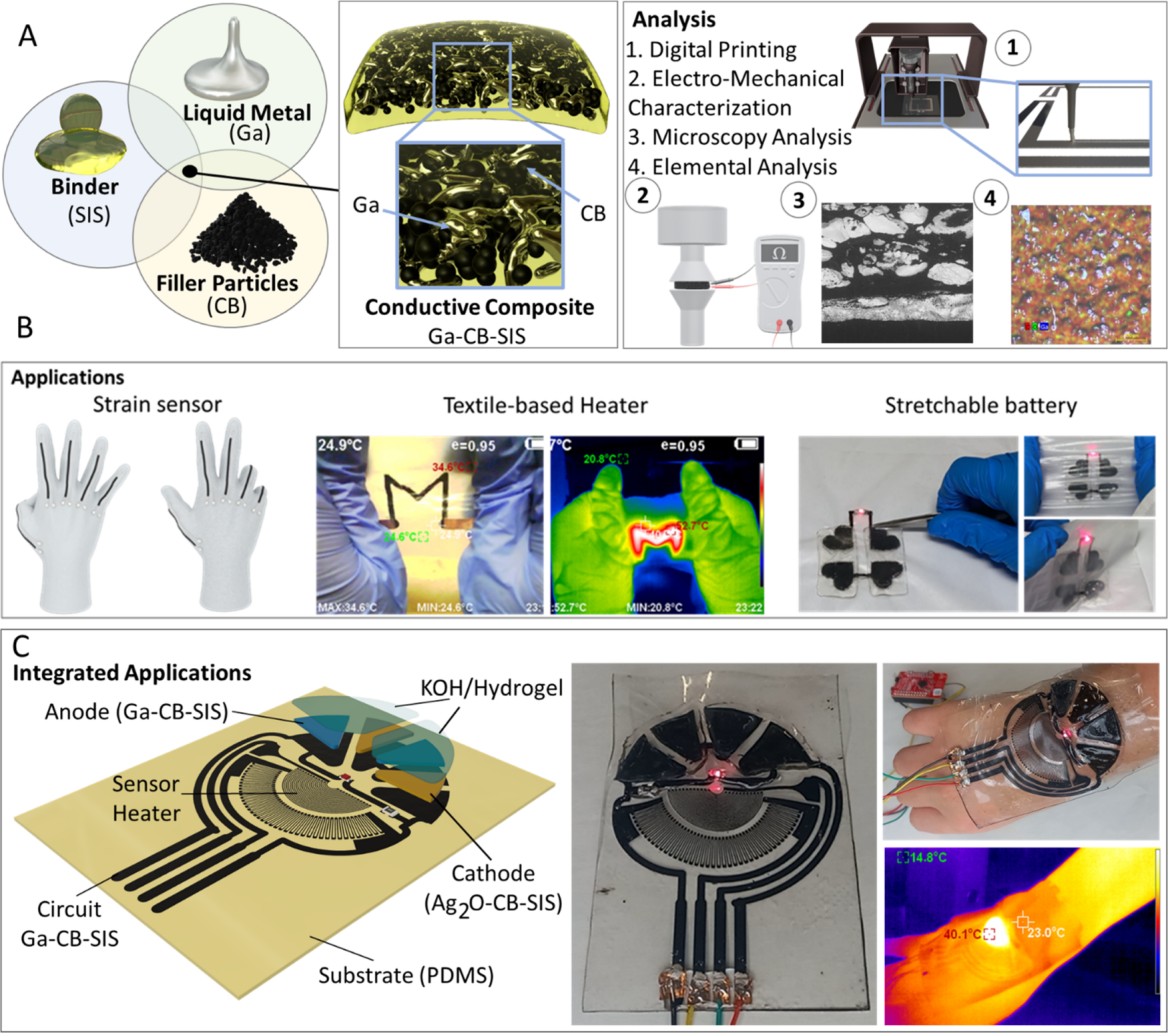
Ga–CB–SIS composite: (A) constitutional materials, characterization, 3D printing; (B) applications; and (C) fabrication of an integrated sensor–heater–battery system.

Figure 1 demonstrates the central concept of the Ga–CB–SIS composite used for various applications, including heated textiles, strain sensors, and thin-film batteries. Here we study and optimize various processes, including synthesis, digital printing, and recycling of the Ga–CB–SIS composite.

Considering that many wearable electronic patches are disposable due to hygiene reasons, it is important to move toward a circular economy. Here, we demonstrate simple and efficient recovery of the Ga particles from circuits through a straightforward, simple, cost-effective, and environmentally friendly approach, using a deep eutectic solvent (DES).^32^

## Results and Discussion

**Figure 2:**
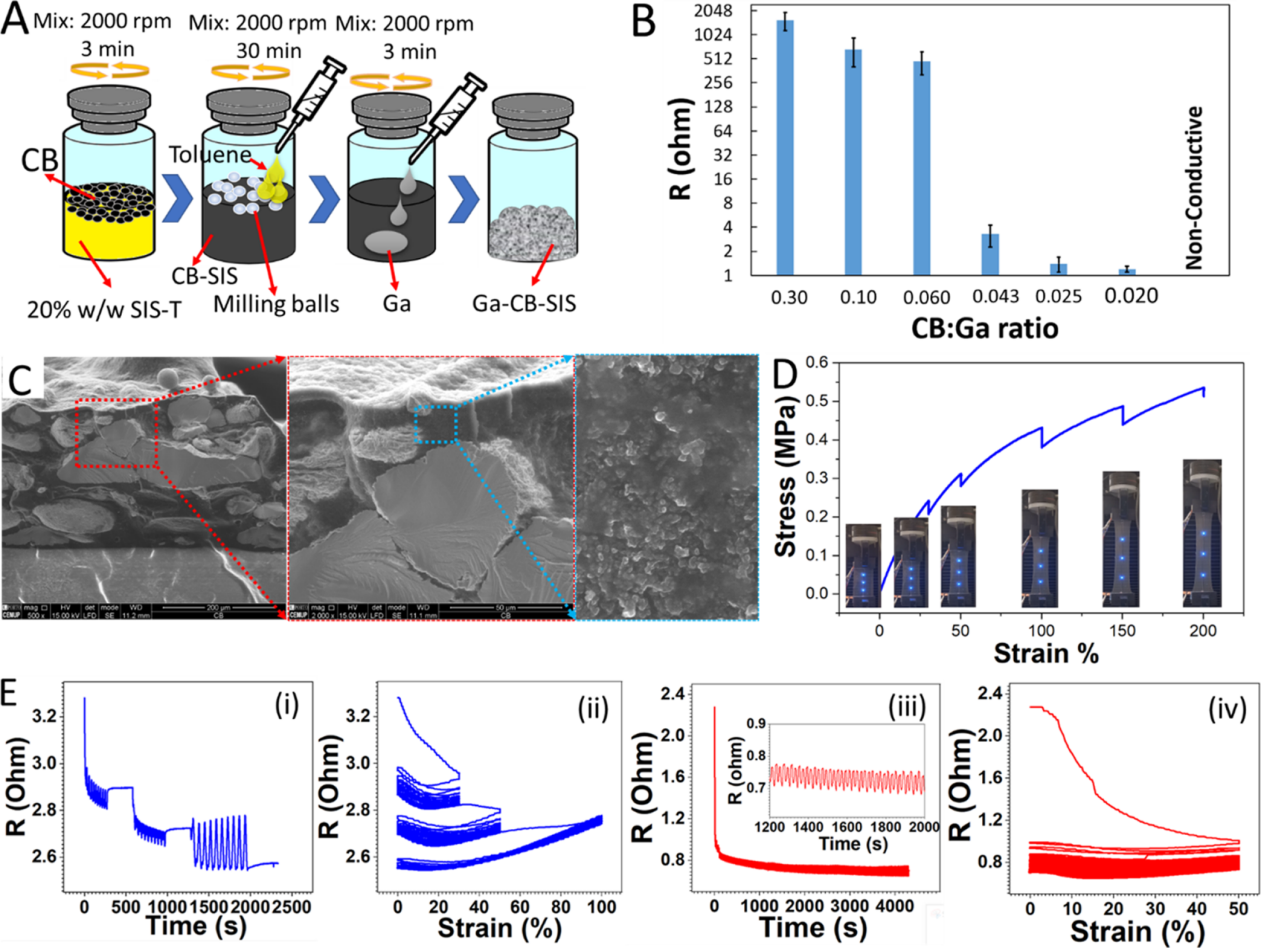
(A) Schematic of the preparation process of the Ga–CB–SIS composite. (B) Resistance of Ga–CB–SIS composite vs different CB/Ga ratio. (C) Cross-sectional scanning electron microscopy (SEM) images of the Ga–CB–SIS composite. (D) Printed trace with light-emitting diodes (LEDs) subjected to different percentages of strain (Max strain ∼200%). (E) Electromechanical characterization of Ga–CB–SIS composite: (i) resistance vs time, (ii) resistance vs strain (30, 50, and 100%) cycling for 10 times, (iii) resistance vs time, and (iv) resistance vs strain 100% cycling for 1000 cycles. In (C), (D), and (E), the CB/Ga weight ratio was 0.043.

Previous efforts on making LM-based composites through mixing LM with elastomers resulted in printable composites. However, these composites were not immediately conductive and required mechanical sintering,^23,24^ where the mechanical sintering cannot be used for the scalable fabrication of high-resolution circuits. Here, CB was used as an additive due to its low cost, low density, and high surface area properties. Generally, carbon-doped elastomers reach the percolation threshold for electrical conductivity at a significantly lower weight percentage, compared to metallic fillers such as Ag. Here, our objective is to identify the optimal mixing strategy and composite that offers better electrical conductivity, resilience to strain, and printability. We first fabricated an LM-free CB–SIS composite with different CB/SIS ratios (1:1, 1:2, and 1:3) and studied their electrical, and electromechanical properties at 30% mechanical strain (Figure S1). At the CB/SIS weight ratio of 1:1, the composite cracks rapidly after deposition, resulting in a high initial resistance of ∼24 kΩ and poor mechanical behavior when subject to mechanical strain. For CB/SIS weight ratios of 1:2 and 1:3, the electromechanical properties of the composite were improved, yet the initial resistance was still in the kΩ range (Figure S1B,C). Indeed, the mixture of Ga with SIS lacks conductivity without mechanical sintering.^3,23^ Conversely, the blending of CB with SIS results in conductivity, but its electromechanical properties are limited, as depicted in Figure S1. This implies that its conductivity is compromised after experiencing a moderate mechanical strain. When Ga is mixed with CB, the composite attains conductivity, but it lacks digital printability, being confined to a pasty consistency. Hence, by incorporating a small quantity of CB into the Ga–SIS composite, we achieve an ink that is both stretchable and conductive, while also being digitally printable. This allows us to create a composite that is not only stretchable and conductive but also compatible with digital printing methods. To achieve this aim, Ga–CB–SIS was synthesized by mechanical mixing of CB with SIS solution, followed by adding Ga LM into the mixture (Figure 2A). The inclusion of the liquid metal particles into the CB–SIS composite improves both the mechanical and electrical behavior of the samples due to the fluidic nature and high electrical conductivity of the Ga LM particles. To study and optimize the composite, we synthesized composites with different CB/Ga ratios. At a CB/Ga ratio of 0.3, the results revealed that the initial resistance remained in the kΩ range, rendering the composite unusable for most applications. An excessive amount of carbon in the composite results in agglomeration of CB, and thus poor percolation. We further decreased the CB/Ga ratio (Figures 2B and S2i). When reaching the CB/Ga weight ratio of 0.06, the initial resistance significantly reduces to 0.2 kΩ, but it is not yet enough for digital circuits. However, these samples demonstrate more stable behavior when subject to mechanical strain as shown in Figure S2ii,iii. As can be seen in Figures 2B and S2A(iv) by further decreasing the CB/Ga ratio was increased to 0.043, the initial resistance remarkably declined to ∼4 Ω (Figure 2B). Further reducing the CB/Ga ratio to 0.025 and 0.02 further improves the resistance to <1 Ω. However, this Ga-rich composite lacks mechanical integrity, and Ga droplets can leak out of the composite, and aggregate, resulting in a nonuniform composite. CB has two roles in the composite. Percolating between gallium particles to form a sinter-free conductive composite, and to make a matrix of particles that hold gallium droplets within. It appears that a CB/Ga ratio of ∼0.04 provides the best combination of electrical conductivity, and mechanical integrity in the composite. Figure S2 shows the electrical resistance of the CB/Ga ratios of 0.3, 0.1, 0.06, and 0.043, 10 cycles of 30, 50, and 100%. As can be seen, at CB/Ga ratios of 0.3 and 0.1, the electrical resistance is very strain sensitive. Reducing this ratio to 0.06 results in a reduced electromechanical coupling and stable behavior over multiple strain cycles.

Figure S3 shows the SEM-SE images of the Ga–CB–SIS composite (CB/Ga weight ratios of 0.3, 0.1, 0.6, and 0.043). Before the election of CB as the filler, we studied the effect of different types of carbons, including graphene oxide (GO), graphite (Gr), multiwall carbon nanotube (MWCNT), Super P conductive carbon (SPCC), and carbon black (CB) to synthesize the Ga–C–SIS composite. Figures 2C and S4 show the cross-sectional SEM-BS images of these composites (maintaining carbon/Ga weight ratio at 0.043). Figure 2D shows the stress–strain curves of the samples (CB/Ga weight ratio 0.043) when subjected to 200% strain. It also shows an LED-integrated circuit fabricated using Ga–CB–SIS composite under strains of 30–200% (Figure 2D). We expected better electromechanical properties for samples with GO, and CNTMW due to their high aspect ratio, but according to Figures 2E and S4, the Ga–SPCC–SIS and Ga–CB–SIS composites presented better electromechanical properties. However, when it comes to electromechanical properties, the Ga–CB–SIS composite presented higher stretchability compared with the Ga–SPCC–SIS composite. After 10 cycles of 30% strain, Ga–SPCC–SIS lost its electrical conductivity. However, the Ga–CB–SIS composite could withstand repetitive cycles of 100% strain (Figure 2E). The SEM images and color map of the Ga–CB–SIS composite (CB/Ga weight ratio of 0.043) is given in Figure S5, for both cases of the pristine sample, and after 10 cycles of strain–stress test.

As for the mixing procedure, obtaining a homogeneous mixture was challenging, when using planetary mixers. Carbon often agglomerates rapidly, even before mixing, thus resulting in a nonhomogenous composite. Prior sonication of CB could slightly improve the composition, yet all composites produced through the above techniques resulted in composites with a sheet resistance in the kΩ/sq range. After experimenting with various mixing techniques and times, we found that simultaneous mixing and ball-milling can achieve the best results. This is performed by adding yttria-stabilized zirconia balls to all samples during mechanical mixing in a planetary mixer. This resulted in a homogeneous composition at the CB/Ga ratio of 4.3% that is digitally printable and obtains a sheet resistance of ∼4 Ω, thus permitting the use of this composite for multiple applications including low-resistance heaters, batteries, and electrical interconnects. As will be discussed, for wearable heaters, the low resistance of the trace is critical to reduce the input voltage. Similarly for digital circuits, it is vital to have low-resistance traces.

### Digital Printing

**Figure 3:**
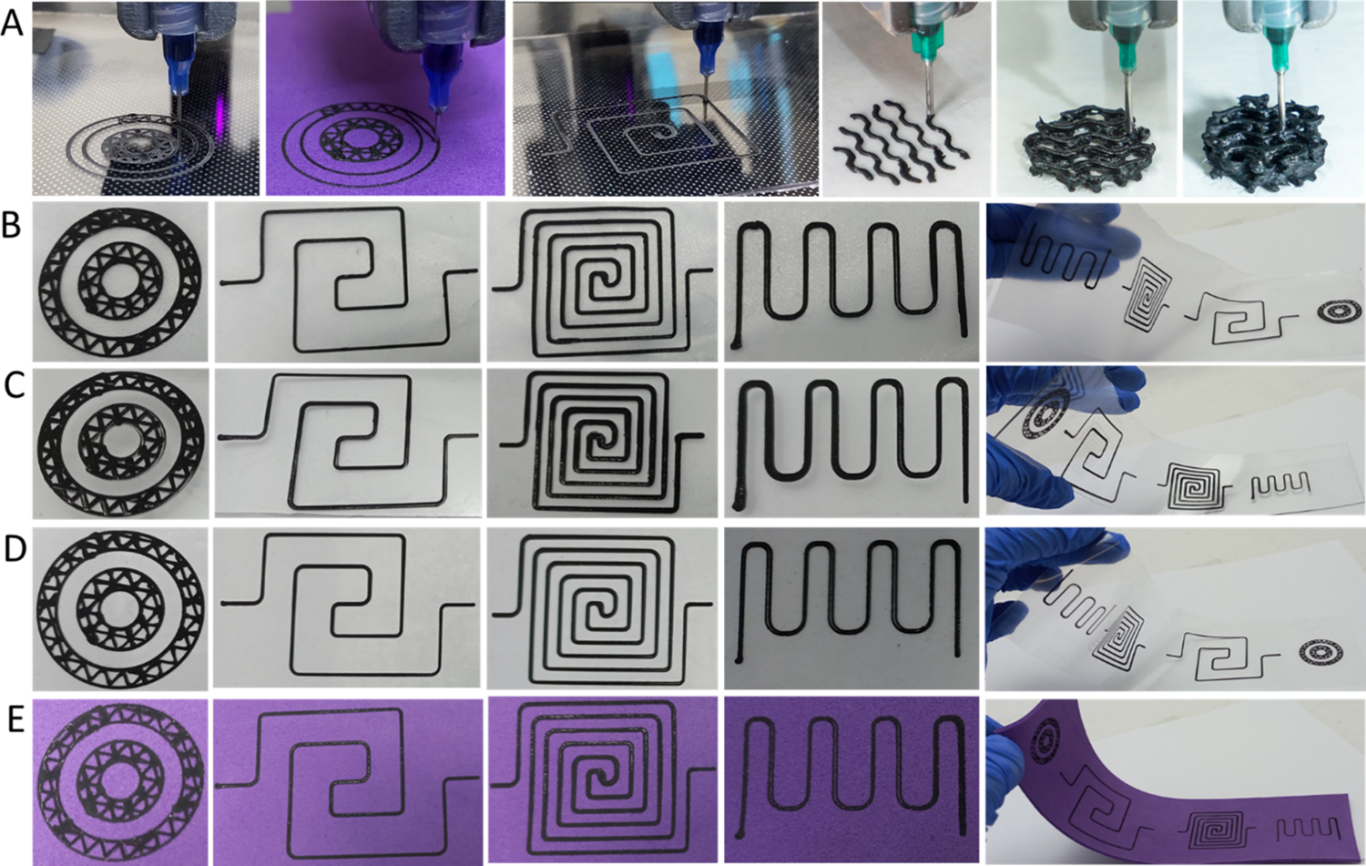
2D and 3D digital printability of the Ga–CB–SIS composite ink over different substrates. (A) Digital printer, 2D and 3D printing state over different substrates, (B) TPU, (C) polycarbonate sheet, (D) acetate sheet, and (E) foam.

Additive manufacturing, encompassing both two-dimensional (2D) and 3D printing, has emerged as a versatile technique for various applications. While liquid metal-based electronics have garnered significant attention, the precise patterning or printing of Ga-based alloys has posed challenges owing to their low viscosity and suboptimal adhesion properties.^12^ In this study, we demonstrate additive manufacturing of the Ga–CB–SIS composite through an accessible extrusion printing process. Figure 3, Video S1, and Video S2 illustrate the 2D and 3D printing states of the as-fabricated ink on diverse substrates, including thermoplastic polyurethane (TPU), polycarbonate sheet, acetate sheet, and foam. The composite ink exhibits a smooth printing behavior and can be printed smoothly over various substrates thanks to its excellent adhesion. This is thanks to the hyperelastic SIS binder in the composite, which has strong adhesion properties to various substrates. Moreover, the composite’s sinter-free nature provides the freedom to print on a wide range of substances, including heat-sensitive plastics and foams. Moreover, Figure S6 indicates the printing states of a benchmark, such as lines with different widths and spacing with their corresponding resolution. A minimum line spacing of 400 μm and a line width of 510 μm could be obtained. See the Supporting Information to find more information about printing parameters. Additionally, the rheological properties of the composites were also considered and are presented in Figures S7 and S8.

### Healing the Damaged Circuits

In cases where circuits produced by the Ga–CB–SIS composite are mechanically damaged, they can be efficiently repaired through a simple solvent vapor exposure setup. Video S3 shows the self-healing behavior of the Ga–CB–SIS composite. The circuit is deeply cut using a knife. Circuit repair is performed through exposure of the circuit to the polymer solvent in a closed chamber.

We utilize styrene isoprene rubber (SIS) reversible block copolymers (BCP) both as the elastomeric binder in the ink and as the substrate material. SIS is a thermoplastic triblock copolymer with both permanent chemical cross-links and physical cross-links. The isoprene sequences chemically cross-linked form longer chains with polystyrene blocks at their ends. These polystyrene blocks aggregate into small domains through physical cross-links. As a result, SIS BCP exhibits valuable properties, including reversible phase-shifting and rapid solubility. Unlike many polymers that only soften upon solvent contact, SIS can soften when subjected to solvent vapor, depending on the duration and intensity of exposure. When the circuit is exposed to solvent vapor, the polymer volume expands, weakening the physical cross-links between the polymer chains. This allows for the reconnection and rearrangement of the polymer chains, effectively self-repairing the cut area. Additionally, the presence of Ga particles in the Ga–CB–SIS composite plays a crucial role in the healing process of the damaged circuit. As the SIS evaporates, the fluidic nature of the Ga particles enables them to move and rejoin. Thus, the synergistic effect of the reversible behavior of the SIS polymers and the fluidic behavior of the Ga particles contribute to the repair of the damaged section of the circuit.

The self-repair mechanism relies on the fluidic nature of LM droplets and the unique properties of the SIS polymer. Incorporating LM particles and the SIS polymer throughout the composite is pivotal for the complete healing of damaged circuits. The toluene-vapor treatment triggers the expansion of polymer bonds, facilitating polymer chain reconnection and circuit repair. During SIS–toluene treatment, the polymer transforms into a gel-like state, allowing LM particles to reconnect within the matrix, establishing conductive pathways, and enhancing mechanical strength.

### Applications

The Ga–CB–SIS composite developed in this work can be used as electrical interconnects, sensors, heaters, and energy storage electrodes, making it possible to develop various applications by using the same material. In this section, we demonstrate some of the applications.

#### Stretchable Strain Sensor

**Figure 4:**
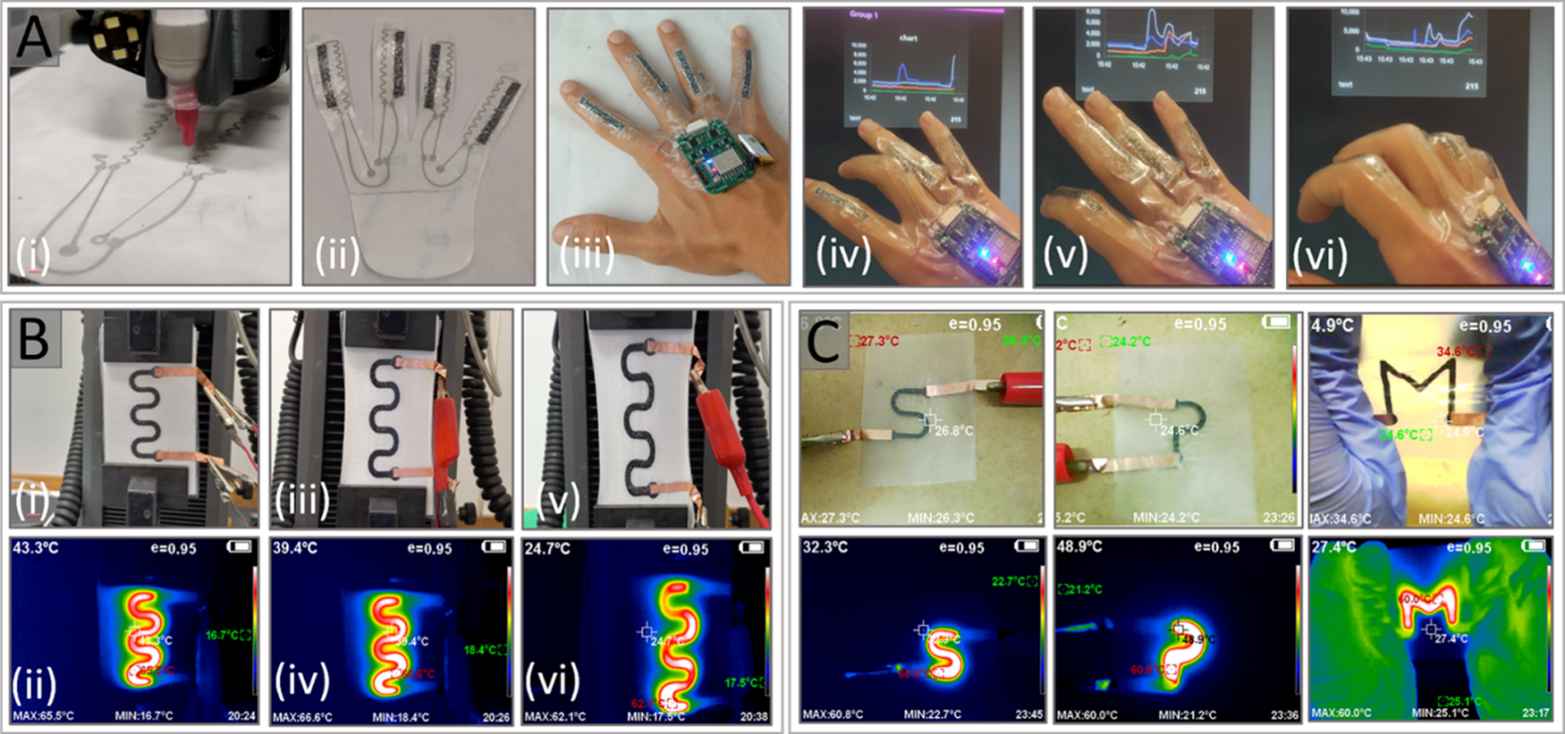
(A) (i) Digitally printing the interconnections for the strain sensor, (ii) full printed interconnections, (iii) wearable patch and acquisition board applied on the volunteer’s hand, and (iv–vi) performance of strain sensor with bending the fingers. (B) e-textile heater (i) and (ii) original state, (iii) and (iv) under 30% strain, (v) and (vi) under 50% strain. (C) Thermal image of different printed patterns.

Soft strain sensors have demonstrated their potential in wearable monitoring and soft robotics as sensory skins.^33,34^ To handle multiscale and dynamic deformations of human organs, strain sensors must maintain their electrical conductivity when they are subject to strain cycles. Here, the main advantage of Ga–CB–SIS is that it can be easily printed through extrusion printing. This permits the development of tailor-made wearable devices per user. Here we show that four strain sensors are printed in a wearable patch that is specifically designed to the size of the volunteer’s hand. This is used for the detection of a hand gesture through a simple digitally printed patch. Figure 4A and Video S4 show the performance of the strain sensor.

#### Textile-based Heater

Ventilation, and air conditioning (HVAC) systems consume about one-third of total energy and they are responsible for the emission of 30% of greenhouse gases in the world.^35−38^ One method to reduce the effect of heating systems on our environment is followed by moving toward the objective of “Heat the person, not the room”. Therefore, heating the textile through Joule heating received increasing attention for application in garments and the seats of electric cars to save energy. This can be done by printing or mounting a heater element on the textile. Ideally, such elements should be thin and soft to maintain the wearable comfort of the textile. At the same time, the printed heater circuit should have a self-healing property to withstand strain cycles that are applied to the textile due to human motion or due to thermal expansion. To provide joint or muscle thermotherapy while exercising, wearable heaters must simultaneously have great stretchability and dynamic stability.^39^ Here, we report that Ga–CB–SIS conductive composite can be easily adopted as ink for printed heaters.

The Joule heating formula is given by *Q* = *I*^2^*Rt*, or *Q* = *V*^2^*t*/*R*, in which *Q* is the amount of heat generated expressed in Joules, *I* is the electric current in Ampere, *R* is the electrical resistance of the circuit in Ohms, *V* is the electrical voltage, and *t* is the time that the current is allowed to flow in the circuit in seconds.

This formula can be as well written as *Q*/*t* = *V*^2^/*R*. That is, the heat delivered through joule heating in a fixed amount of time, inversely relates to the electrical resistance. Therefore, to rapidly deliver heat to a system, usually low resistance of the composite is preferred, which is the case for Ga–CB–SIS composite. Also, for wearable applications, it is generally preferred to maintain low voltages for safety.

In this work, we printed two layers of a Ga–CB–SIS composite (CB/Ga ratio of 0.043) over a textile. Before printing, we applied an ultrathin film of ∼60 μm TPU film on the textile to ensure good printability. The setup was sealed with another layer of TPU by using a heat press. The initial resistance of the sample (3 × 120 mm^2^) at 0% strain was 12 Ω. When a voltage of 2 V was applied for 30 s the temperature reached 65 °C. Under 30% strain, the resistance decreased to 8 Ω, and to maintain the temperature at ∼65 °C, the applied voltage decreased to 1.3 V. At 50% strain, the resistance was 3 Ω, and with 0.85 V of applied voltage, the temperature reached 62 °C (Figure 4B). In all cases, a voltage of less than 2 V is enough to reach a comfortable temperature in a few seconds. Thanks to the possibility of digital printing, we can print the Ga–CB–SIS composite with different patterns to tailor-made the shape of the wearable heater for a user and to adjust its electrical resistance for the desired voltage. Figure 4C shows the digital photo and infrared temperature distribution images of multiple patterns of the Ga–CB–SIS composite.

The low-voltage operation of the heater is critical and depends on the resistance of the ink, as known from the *Q*/*t* = *V*^2^/*R*. Therefore, reaching a sheet resistance in the Ω range is critical for wearable heaters. The Ga–CB–SIS composite has therefore excellent properties as an ink for thermal heating. It permits rapid heating of the textile with low voltages, it can withstand mechanical strain, and it is digitally printable, thus permitting rapid prototyping of tailor-made wearable heaters.

### 3D-Printed Stretchable Liquid Gallium Battery

#### Toward Stretchable Ga–Ag_2_O Battery

The development of mesoscale electrochemical energy storage devices is blossoming due to the increasing need for lightweight, integrable, flexible, customized, and miniaturized electronics.^40−42^ Miniaturized power sources, such as thin-film printed batteries, are currently gaining popularity due to rapid advances in wearable electronics.^43−45^ However, the development of techniques to quickly design and fabricate a tailor-made thin-film battery with a particular structure is challenging due to the complex and multimaterial architecture of the battery. Adding to this the need for making the battery stretchable makes it further challenging, as this requires the use of a fully stretchable architecture of materials, including current collectors (CCs) and electrodes.

Additive manufacturing (AM), also known as three-dimensional (3D) printing, is a rapid, flexible, and affordable method for fabricating electrodes with high mass loadings for rechargeable miniaturized batteries.^43,46,47^

AM enables the rapid fabrication of tailor-made printed batteries. In comparison to conventional methods, 3D printing offers significant control over the geometrical shape of the electrodes, rigidity, porosity, and thickness, allowing for the creation of complex architectures as desired.^48,49^

When considering wearable patches and textiles, the possibility of direct digital printing of the battery on the patch brings significant advantages over the traditional method of integrating a coin-cell battery. It results in wearable devices that are thinner and more mechanically integrated, especially in cases where mechanical strain should be applied. Moreover, the size and mass loading can be precisely controlled per application.

Recently the use of low-melting-temperature metals including gallium and its alloys as an additive to lithium batteries or as a stand-alone battery electrode was reported in a few works.^50−53^

**Figure 5:**
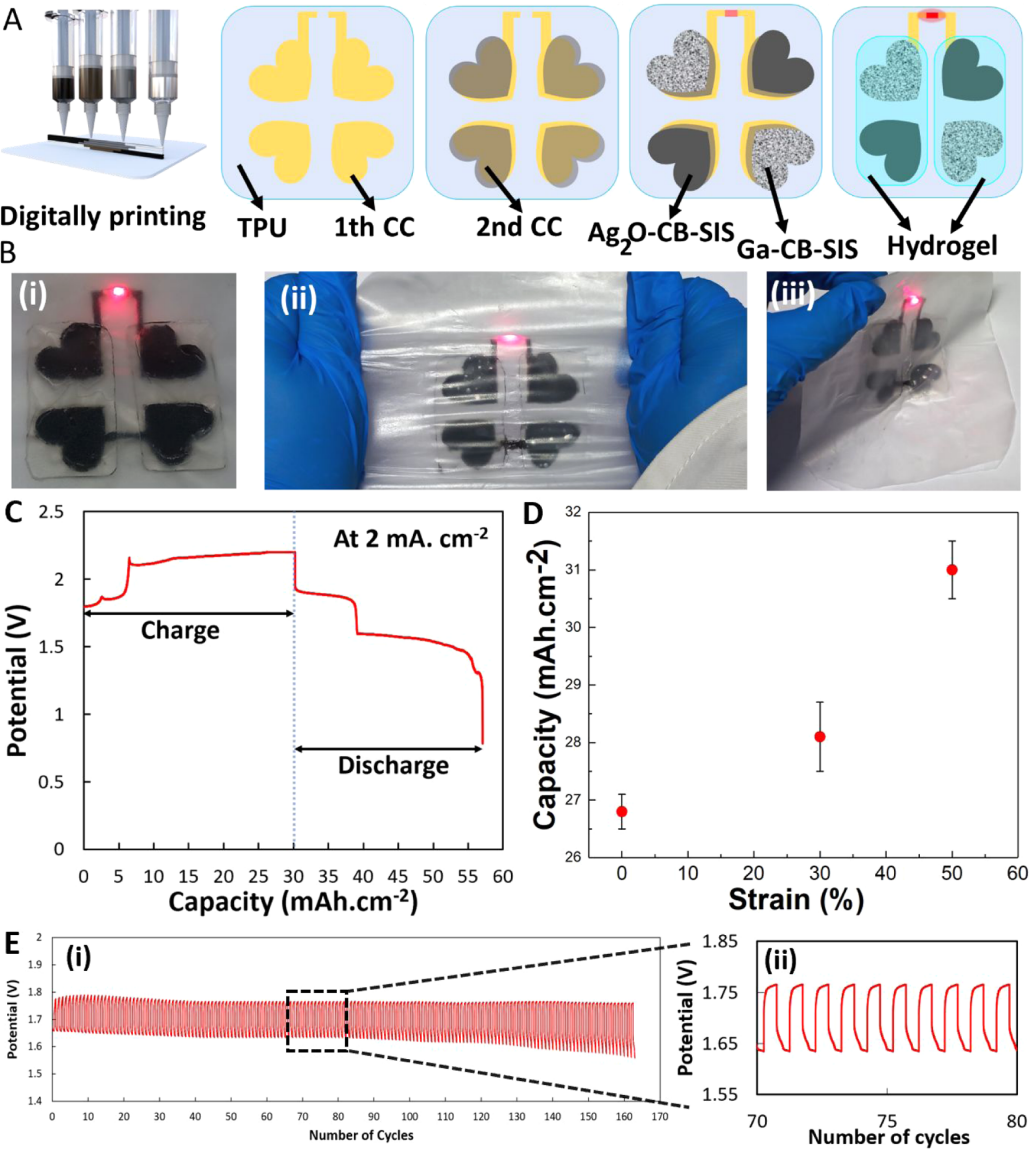
(A) Printing steps of the Ag_2_O–Ga battery, Ga–CB–SIS as the anode, and Ag_2_O–CB–SIS as the cathode. (B) (i) Original state of the digital printable behavior of the assembled Ag_2_O–Ga battery, the red LEDs are in the on-state, (ii) stretching and (iii) folding states, as is also shown in Video S5. (C) Potential profile of the Ag_2_O–Ga stretchable battery at 2 mA·cm^–2^. (D) Capacity of the battery under 0, 30, and 50% strain at 2 mA·cm^–2^. (E) Galvanostatic discharge–charge cycle performance of the battery at 0.8 mA·cm^–2^: (i) all cycles and (ii) the cycles 70–80 s.

Here we show that the Ga–CB–SIS composite can be used as well as an anode electrode to form a fully printed thin-film silver gallium battery. Figure 5A shows a schematic of the material architecture of the battery. All layers that were used as first and second current collectors (CCs), active materials (anode and cathode), and LED interconnections are digitally printable. Therefore, the full battery architecture can be digitally printed with custom size, shape, and mass loading depending on the application. Ag–EGaIn–SIS composite is used as the first CC because of its high conductivity (7.02 × 10^5^ S·m^–1^) and outstanding stretchability (>1700%).^29^ A composite of carbon black and SIS (CB–SIS) was printed as the second current collector to prevent an unwanted reaction between the first CC and the hydrogel electrolyte. We did not include any metallic filler in the second CC, to avoid any redox reaction between the electrode and the electrolyte. Then, we digitally printed the anode (Ga–CB–SIS) and the cathode (Ag_2_O–CB–SIS). This constitutes a Ag–Ga battery. All four composites include the hyperelastic SIS binder to guarantee mechanical integrity between layers. As an electrolyte, we soaked acrylamide-sodium alginate hydrogel in 35% w/w KOH solution and applied this over electrodes at the end. Finally, the battery was sealed with a layer of TPU through a heated iron mold that efficiently sealed the borders. Figure 5B and Video S5 show the battery that lights on the LED light under mechanical strain and twisting. An example of a charging and discharging cycle at a constant current of 2 mA·cm^–2^ is shown in Figure 5C. Two characteristic plateaus of the silver oxide at ∼1.8 and 1.6 V are visible. The areal capacity of the battery is 26.86 mAh·cm^–2^, which is higher than previously reported stretchable batteries.^50,51,54−56^ In one experiment, we discharged the battery at 2 mA·cm^–2^ while being subject to mechanical strain. Figure 5D shows the results for the maximum capacity of the battery under 0, 30, and 50% strain (five samples were used for each test). Referring to Figure 5D, the areal capacity of the battery slightly increased under strain. The enhanced capacitance of the liquid metal composite under strain may be attributed to its increased conductivity of the CCs, resulting from the rupture of the liquid metal droplets’ shells, as described in our prior publications.^3,28^ Moreover, this can be related to the improved electrode–hydrogel interface as uniaxial strain generates a compressive force between the two materials. Figure 5E shows the cyclic performance of the battery charged and discharged at 0.8 mA·cm^–2^.

### Versatile Composite

#### As a Stretchable and Integrated Sensor–Heater–Battery System in Wearable and Recyclable Electronics

The Ga–CB–SIS composite can be used to fabricate a stretchable battery, sensor, or textile-integrated heater. To demonstrate this, we designed and implemented a circuit that integrates a heater and a strain sensor that is powered by a stretchable Ga–Ag_2_O battery in a single patch (Figure 1C). The Ga–CB–SIS composite was laser patterned to the desired shape, and the Ga–Ag_2_O battery was connected to the sensor and element. Consisting of two batteries in series, the system satisfies a 3 V input requirement for the electronics. These batteries are connected to the sensor to supply the required power. The sensor element can work as a heater, but as the Ga–Ag_2_O battery is not able to supply the required current that is needed to the heater, the heater is connected to an external power supply. The circuit, connected to the BCP patch, incorporates a low-power Bluetooth communication chip (Cypress Semiconductor) for wireless data transmission, allowing the patch to send strain data wirelessly to a PC via Bluetooth. Video S6 shows the performance of the integrated sensor, heater, and Ga–Ag_2_O battery. Figure S9A,B(i) shows a schematic diagram of the working principle and main components of the PCB. Figure S9A(ii) shows the collected results of the strain sensor, and Figure S9B(ii) is the thermal image of the heater element that reached 40 °C when a voltage of 4 V was applied.

#### Recycling of the Ga LM through a Deep Eutectic Solvent (DES)

**Figure 6:**
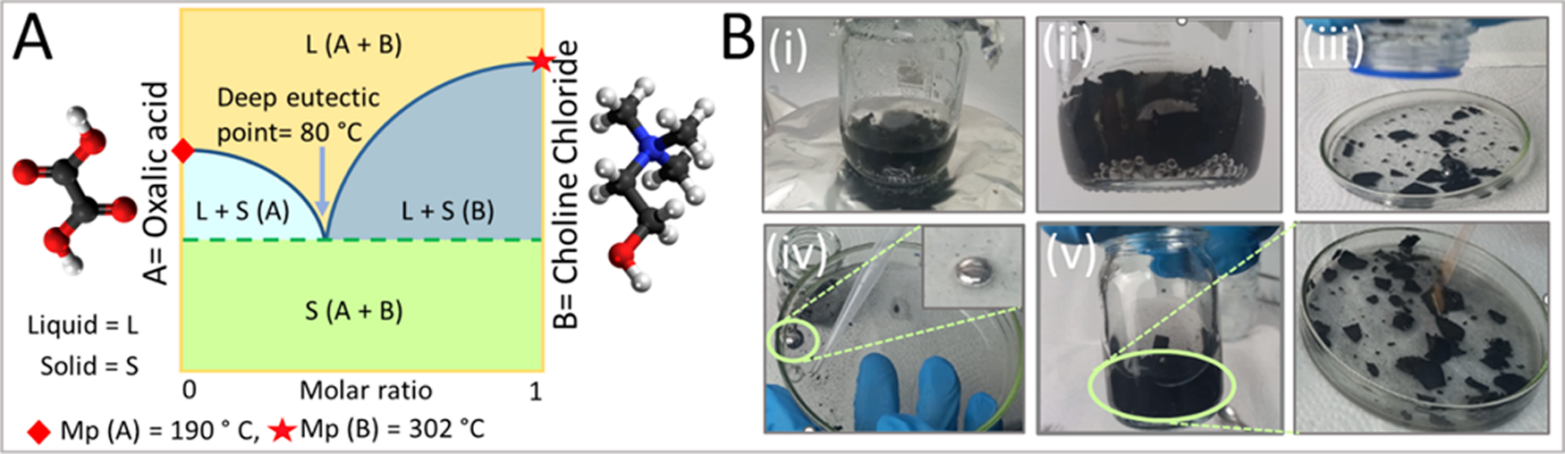
(A) Schematic presentation of DES preparation with oxalic acid and choline chloride, molar ratio 2:1, and (B) recycling of the Ga–CB–SIS composite ink e-waste via the DES method.

Gallium (Ga) is a valuable metal with unique properties, that has applications in electronics, semiconductors, and solar cells. The demand for gallium has been constantly increasing in the past years. In previous works, sodium hydroxide, and hydrochloric acid were shown to be able to recycle Ga or EGaIn LMs from electronic wastes.^24,57−61^ Here, we extend LM extraction with a new technique using deep eutectic solvents (DES). DES has been used in the recycling process of transition metals^62,63^ and is considered environmentally friendly, an alternative to conventional organic solvents.^32^ Inspired by these works, we developed a technique for recycling the gallium from the composite with an efficiency of ∼98%. This is performed by dissolving the printed Ga–CB–SIS ink e-waste in a deep eutectic solvent (DES) composed of choline chloride and oxalic acid (OA) with a ratio of 1:2 (Figure 6A). Figure 6B shows the recycling process. The e-waste was put in the as-prepared DES, and most of the LM particles were collected under a magnetic stirring process (Video S7). This was followed by extra mechanical mixing to extract the remained LM particles; some extra LM was extracted. Using choline chloride (ChCl) as hydrogen-bonded-acceptor (H_BA_) and oxalic acid (OA) as hydrogen-bonded-donor (H_BD_), deep eutectic solvents (DESs) with a molar ratio of 1:2 was prepared. The mixture was heated to 80 °C on a magnetic stirrer for about 15 min to obtain a homogeneous solvent. The H_BA_ and H_BD_ interaction in DES causes a decline in the mixture’s freezing point in comparison with the initial freezing point of each component,^64^ containing an assortment of anionic and/or cationic species.^65^ To explain one possible mechanism to extract Ga LM from the Ga–CB–SIS composite e-waste we hypostasize that similarly with HCl solution^66^ and HNO_3_ solution,^67^ the surface tension of the Ga liquid metal immediately increases when the Ga–CB–SIS composite e-waste is exposed in the as-prepared DES and it becomes easier to break away from the elastomer matrix. This means that the Ga droplets are extracted by carboxylic acids forming dimeric rings strongly linked by intermolecular hydrogen bonding between the OH and C–O groups.^68^ In the recent work,^68^ it has been stated that the OH stretching associated with OH–Cl– in ChCl with related H_BD_. The formed DES through ChCl and oxalic acid is a strong acid with a pH of 0.5. So, it can be used as an acid catalyst to transform the hydroxy group in carboxylate into ester form to remove the oxide layers surrounding the Ga LMs in the composite. In contrast to HCl, the acid formed from DES, in this study, has usefulness as an environmentally friendly and sustainable replacement for conventional such harmful solvents, e.g., HCl and HNO_3_, or as catalysts used in synthetic chemistry.^69^ Overall, the proposed approach allows the recycling of Ga particles from e-waste, increasing the economic and environmental feasibility of these green/sustainable electronics, which are more economically and environmentally viable as a result of the use of DES to recycle LM.

## Conclusions

In this work, we presented a Ga–CB–SIS composite, a digitally printable, sinter-free, and recyclable composite that can be printed on a wide range of substrates and can be used as an electrical interconnect, heater, sensor, and battery electrode. The sinter-free properties open doors to widening the scope of printed electronics for a wide range of plastics, paper, and textiles. This is also important in the move toward greener electronics, especially considering the new generation of biopolymers or biodegradable polymers that are generally more fragile to temperature or mechanical forces. The ability to recover Ga LM using the “deep eutectic solvent” technique with high efficiency further adds to the sustainability of the material. Overall, this composite material has good potential in the field of sustainable electronics.

The Ga–CB–SIS overcomes the limitations of previous LM–CB and SIS–CB composites. Such as the need for mechanical sintering and sensitivity to mechanical strain. The fabrication of a homogeneous and high-conductivity Ga–CB–SIS composite was, however, not trivial and required studying a wide range of carbon-based materials and compositions to guarantee a good trade-off between electrical conductivity, stretchability, and digital printing.

Another unique property of the Ga–CB–SIS composite is that the same composition can be used in various applications such as sensing, heating, and energy storage. This is particularly important in combination with the possibility of 3D printing as it permits direct digital printing of integrated circuits that embed sensing, heating, and energy storage elements. These circuits can be easily tailor-made per user and application thanks to the possibility of digital printing. This is a move toward fully printed devices and eliminates the need for the integration of individual sensors, batteries, and heating elements in wearable devices.

## Materials and Experimental Section

### Materials

All materials, unless stated otherwise, were used as received. Gallium and indium were ordered from Rotometals and Nova Elements, respectively. EGaIn was synthesized by melting and mixing 75.5 wt % of Ga and 24.5 wt % of In at 250 °C for 24 h. Silver Flakes 071 with a particle size of >5 μm were obtained from Technic, Inc. Carbon Black from AlfaAesar (45527) was used in the study. Polystyrene-*block*-polyisoprene-*block*-polystyrene (SIS) with 14% styrene was ordered from AlfaAesar. Silver(I) oxide, >99% (metals basis) was obtained from AlfaAesar (11407). Calcium sulfate, irgacure, acrylamide, methylenebis(acrylamide) (BIS), and sodium alginate were ordered from Sigma-Aldrich.

### Equipment

The mixer employed in this study was Thinky ARE-250. Plastic stencils were cut by using a CO_2_ laser cutter (VLS3.50) for the stencil printing process. All of the composites were digitally printed using our lab-made 3D printer.

### SEM Microscopy and Energy-Dispersive X-ray Spectroscopy (EDS) Analysis

The morphology and microstructure of the composites were analyzed using SEM coupled with energy-dispersive X-ray spectroscopy (EDX) and mapping. The equipment used for this analysis was a Bruker Nano GmbH Berlin, Germany Esprit 1.9 with a Detector type XFlash 410.

### Composite Fabrication

Initially, a mixture consisting of 2.7 g of SIS–toluene 20% and 0.3 g of CB is blended at 2000 rpm for 3 min. Subsequently, an additional 1 g of toluene and 1 g of zirconium balls are added, and the mixture is further blended for 30 min at 2000 rpm. Four equal compositions are prepared. Finally, 1, 3, 5, and 7 g of Ga are added to each composition, respectively, and milled at 2000 rpm for 3 min.

### Hydrogel and Electrolyte

To prepare the hydrogel electrolyte, we dissolved 0.732 g of sodium alginate, 0.006 g of BIS, and 4.5 g of acrylamide in 30 mL of water. The solution was then degassed, and 20 mL of it was transferred to a syringe. In another syringe, we mixed 0.0492 g of irgacure and 0.0646 g of calcium sulfate with 1 mL of water. Using a syringe adapter, we combined the contents of both syringes and cast the resulting mixture onto a glass surface, where it was cured under UV light for approximately 3 h. Finally, we immersed the cured hydrogel in a 35 wt % KOH solution for 24 h.

### Applications Measurement

To evaluate the thermal management system of wearable devices, we initially applied TPU on top of a stretchable textile and subsequently printed the Ga–CB–SIS composite. We sealed the composite with another layer of TPU and used an infrared thermal imager (XEAST XE-33, 320 × 240 resolution, China) to monitor the temperature changes of the DC-powered wearable thermal management circuits.

### Strain Sensor

The acquisition and processing circuit board employs four 24-bit analog-to-digital converters (ADCs) and four digital-to-analog converters (DACs) that are directly governed by the ESP8266 microcontroller. The ESP8266 microcontroller samples four distinct CARBON strain sensors at a frequency of 10 Hz. Subsequently, the microcontroller transmits the collected data wirelessly over a 2.4 GHz WiFi network to an MQTT server that runs on a computer. The MQTT server then generates a webpage that is customized to display the graph of each strain sensor in a comprehensible format.

### Battery Architecture

In this study, we successfully demonstrated the fully digital printing of a stretchable Ag_2_O–Ga battery using a combination of four digitally printable composites (Figure 5). The entire process was carried out at room temperature and did not require any sintering, which makes it compatible with a broad range of heat-sensitive substrates. Our first step involved printing a biphasic stretchable composite, which serves as the primary current collector (CC), using a sinter-free ink composed of Ag flakes, eutectic gallium indium (EGaIn), and styrene isoprene block copolymers (SIS). This stretchable composite has several advantages, including high conductivity, low gauge factor, stable behavior over numerous cycles, and excellent stretchability of up to 600% without any significant loss of conductivity. Unlike the liquid metal itself, the ink dries after deposition and is nonsmearing, which enables the printing of subsequent layers over it. The second CC layer was made up of carbon black (CB) and SIS, which protects the first CC layer from chemical corrosion. The electrodes consisted of a printable Ag_2_O–SIS cathode and a novel printable gallium–carbon–SIS (Ga–C–SIS) anode.

### Integrated Application

Initially, we coated a glass plate with a 10% aqueous solution of poly(vinyl alcohol) (PVA), followed by the application of a layer of the Ga–CB–SIS composite using a rod. After the mixture was allowed to cure, another layer of the composite was added. Subsequently, an accessible IR MOPA laser (1064 nm) was utilized to pattern the desired circuit geometry through complete material ablation from the film (Figure 1). A layer of PDMS was then applied as a substrate over the circuit by using a thin film applicator. The layers were detached from the glass and immersed in a water tank to eliminate the PVA layer. In this stage, cathode and anode active materials were applied by using an orange stencil mask. Then, we connected the BCP patch and LED to the circuit and applied the KOH/hydrogel on the battery’s electrode. The circuit integrates a Bluetooth communication chip (Cypress Semiconductor PSOC4 BLE) for wireless data transmission.

## Supporting Information Available

Electromechanical characterization of CB–SIS composite (Figure S1); electromechanical behavior of the Ga–CB–SIS composite (Figure S2); SEM-SE images and color map of the gallium and carbon black for different CB/Ga weight ratios (Figure S3); C–Ga–SIS composite (CB/Ga weight ratio 0.043) with different types of carbon (Figure S4); SEM-SE images and color map of the gallium and carbon black in the Ga–CB–SIS composite (CB/Ga weight ratio 0.043) (Figure S5); printing states of benchmark lines with different widths and spacing, resolution settings (Figure S6); schematics representing the setup used for extrusion testing (Figure S7); characterization of the ink composites (Figure S8); comparison of previous works and this study based on composite, electromechanical properties, printability, and application (Table S1); and comparison of previous works and this study based on materials, electrical properties, strengths, and weaknesses (Table S2) (PDF)
2D printing states of ink on various substrates (TPU, polycarbonate sheet, acetate sheet, and foam) (Video S1) (MP4)
3D printing states of ink on various substrates (TPU, polycarbonate sheet, acetate sheet, and foam) (Video S2) (MP4)
Self-healing behavior of the Ga–CB–SIS composite (Video S3) (MP4)
Strain sensor performance demonstration (Video S4) (MP4)
Battery lighting an LED under mechanical strain and twisting (Video S5) (MP4)
Performance demonstration of integrated sensor, heater, and Ga–Ag_2_O battery (Video S6) (MP4)
Recycling of Ga LM using a deep eutectic solvent (Video S7) (MP4)
