# Supplementary material for: Gallium–Carbon: A Universal Composite for Sustainable 3D Printing of Integrated Sensor–Heater–Battery Systems in Wearable and Recyclable Electronics
Source: ACS Appl Mater Interfaces. 2024 Jun 15;16(25):32812–23. doi: 10.1021/acsami.4c02706 (PMC11212025; doi:10.1021/acsami.4c02706)
Supplement: Supplementary file 1 — am4c02706_si_001.pdf [file am4c02706_si_001.pdf]

“Supporting Information (SI) for Publication file”

Gallium-Carbon: A universal Composite for Sustainable 3D Printing of Integrated Sensor-Heater-Battery Systems in Wearable and Recyclable Electronics

Elahe Parvini[1], Abdollah Hajalilou[1], João Pedro Gonçalves Vilarinho[1], Pedro Alhais Lopes[1], Miguel Maranha[1], Mahmoud Tavakoli[1*]

1. Soft and Printed Microelectronics Lab, Institute of Systems and Robotics, University of Coimbra, Coimbra, 3030-290 Portugal

***Corresponding author E-mail: mahmoud@isr.uc.pt**

Figure S1. Electromechanical characterization of CB-SIS composite, **A)** shape and size of dog-bon samples, **B)** 1:1, **C)** 1:2, and **D)** 1:3 weight ratio CB: SIS. In all figures A, B, and C: i) Resistance vs. Time, ii) Resistance vs. Strain, iii) Digitally image of dog bone-shaped samples.

Figure S2. Electromechanical behavior of the Ga-CB-SIS composite, **A)** R/R_0 vs. Strain (%) and **B)** R/R_0 vs. Time (s) with different compositions CB: Ga ratios (i) 0.3, (ii) 0.1, (iii) 0.06 and (iv) 0.043 printed over latex substrate.

Figure S3. SEM-SE images and color map of the gallium and carbon black for CB: Ga weight ratio **A)** 0.3, **B)** 0.1, **C)** 0.06, and **D)** 0.043.

Figure S4. The C-Ga-SIS composite (CB: Ga weight ratio 0.043) with different types of carbon, **A)** Graphene oxide, **B)** Graphite, **C)** Multi-wall carbon nanotube, **D)** Super P conductive carbon. In all Figures, (i) Cross-sectional SEM-BS images and (ii) Electromechanical characterization under different strains (30%, 50%, and 100%) for 10 cycles and in the case of Super P conductive carbon under 30% strain for 100 and 500 cycles.

Figure S5. SEM-SE images and color map of the gallium and carbon black in the Ga-CB-SIS composite (CB: Ga weight ratio 0.043), **A)** pristine sample, **B)** after 10 cycles of strain-stress test.

Figure S6. This indicates the printing states of a benchmark, such as lines with different widths and spacing with their corresponding resolution (based on the printer settings shown under the printed images).

Figure S7. Schematics representing the setup used for extrusion testing.

Figure S8. Characterization of the ink composites. Ag-EGaIn-SIS at 0.1 mm/min. Second current collector (CB-SIS) at 1 mm/min. Ag_2O-CB-SIS ink at 0.5 mm/min. Ga-CB-SIS at 0.5 mm/min.

Figure S9. A) Schematics diagram exhibiting the working principle and main components of the Sensor−PCB (i), The recorded results via Bluetooth (Strain (ADC counts) vs. time (s) (ii), **B)** the working principle and main components of the Heater−PCB (i), Thermal image of heater (ii).

Table S1: Characteristics of the previous works compared to this work: Based on the composite, electromechanical properties, printability, and application.

Table S2: Characteristics of the previous works compared to this work: based on materials, electrical properties, and strengths and weaknesses.

Supporting Videos:

Video S1 and **Video S2** illustrate the 2D and 3D printing states of the as-fabricated ink on diverse substrates, including TPU, Polycarbonate sheet, Acetate sheet, and Foam.

Video S3 shows the self-healing behavior of the Ga-CB-SIS composite.

Video S4 shows the performance of the strain sensor.

Video S5 shows the battery that lights on the LED light under mechanical strain and twisting.

Video S6 shows the performance of the integrated sensor, heater, and Ga-Ag_2O battery.

Video S7 shows the recycling of the Ga LM through a deep eutectic solvent (DES).

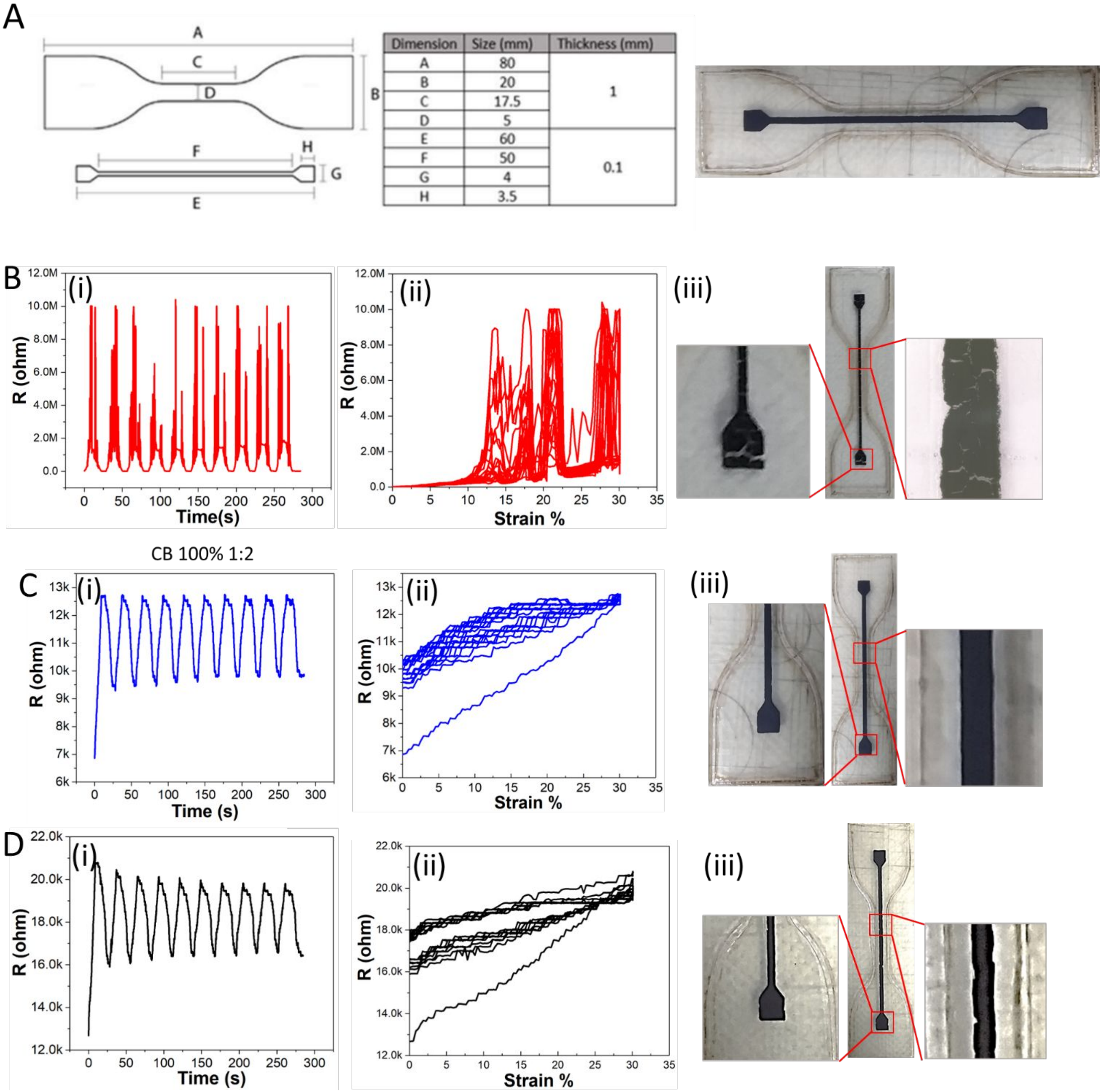

Figure S1. Electromechanical characterization of CB-SIS composite, **A)** shape and size of dog-bon samples, **B)** 1:1, **C)** 1:2, and **D)** 1:3 weight ratio CB: SIS. In all figures A, B, and C: i) Resistance vs. Time, ii) Resistance vs. Strain, iii) Digitally image of dog bone-shaped samples.

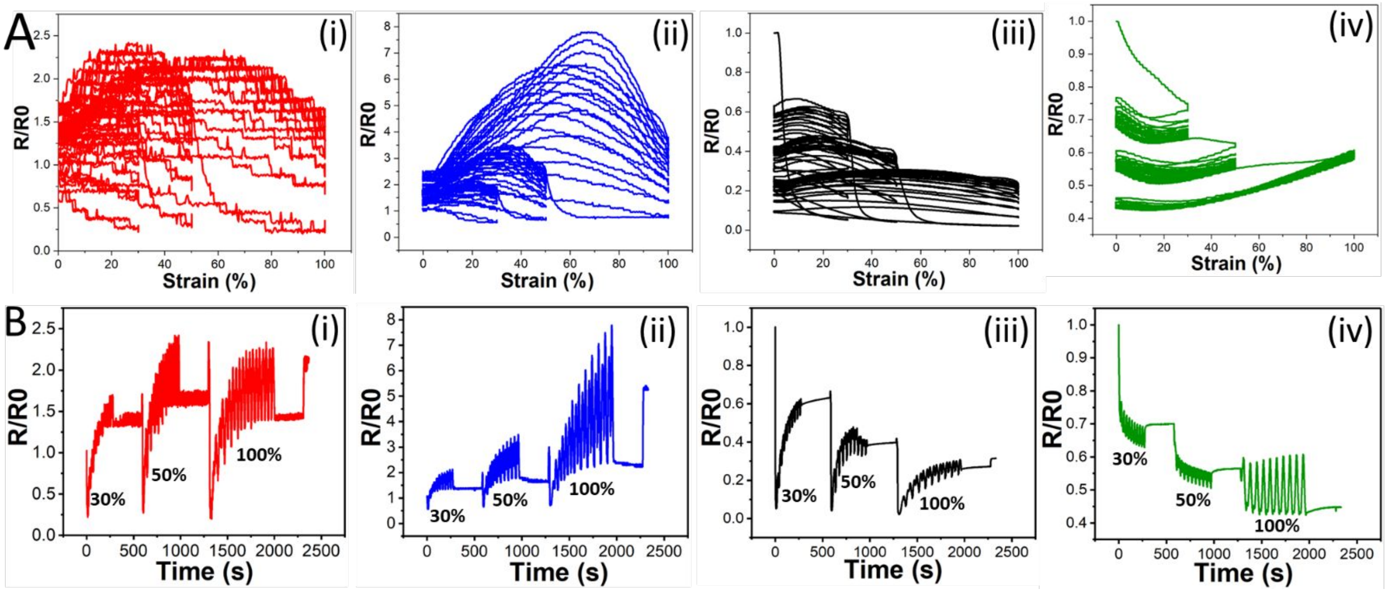

Figure S2. Electromechanical behavior of the Ga-CB-SIS composite, **A)** R/R_0 vs. Strain (%) and **B)** R/R_0 vs. Time (s) with different compositions CB: Ga ratios (i) 0.3, (ii) 0.1, (iii) 0.06 and (iv) 0.043 printed over latex substrate.

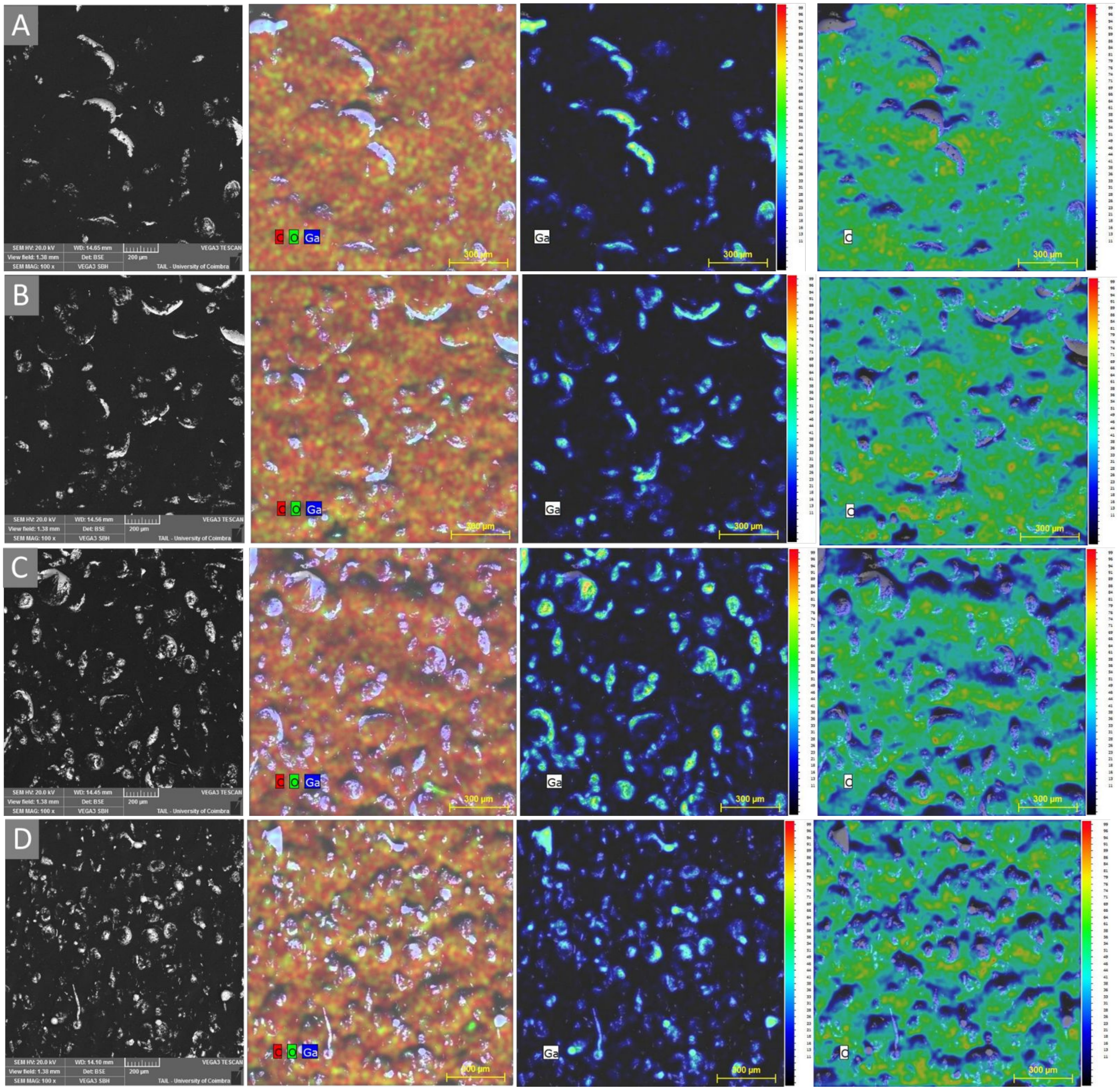

Figure S3. SEM-SE images and color map of the gallium and carbon black for CB: Ga weight ratio **A)** 0.3, **B)** 0.1, **C)** 0.06, and **D)** 0.043.

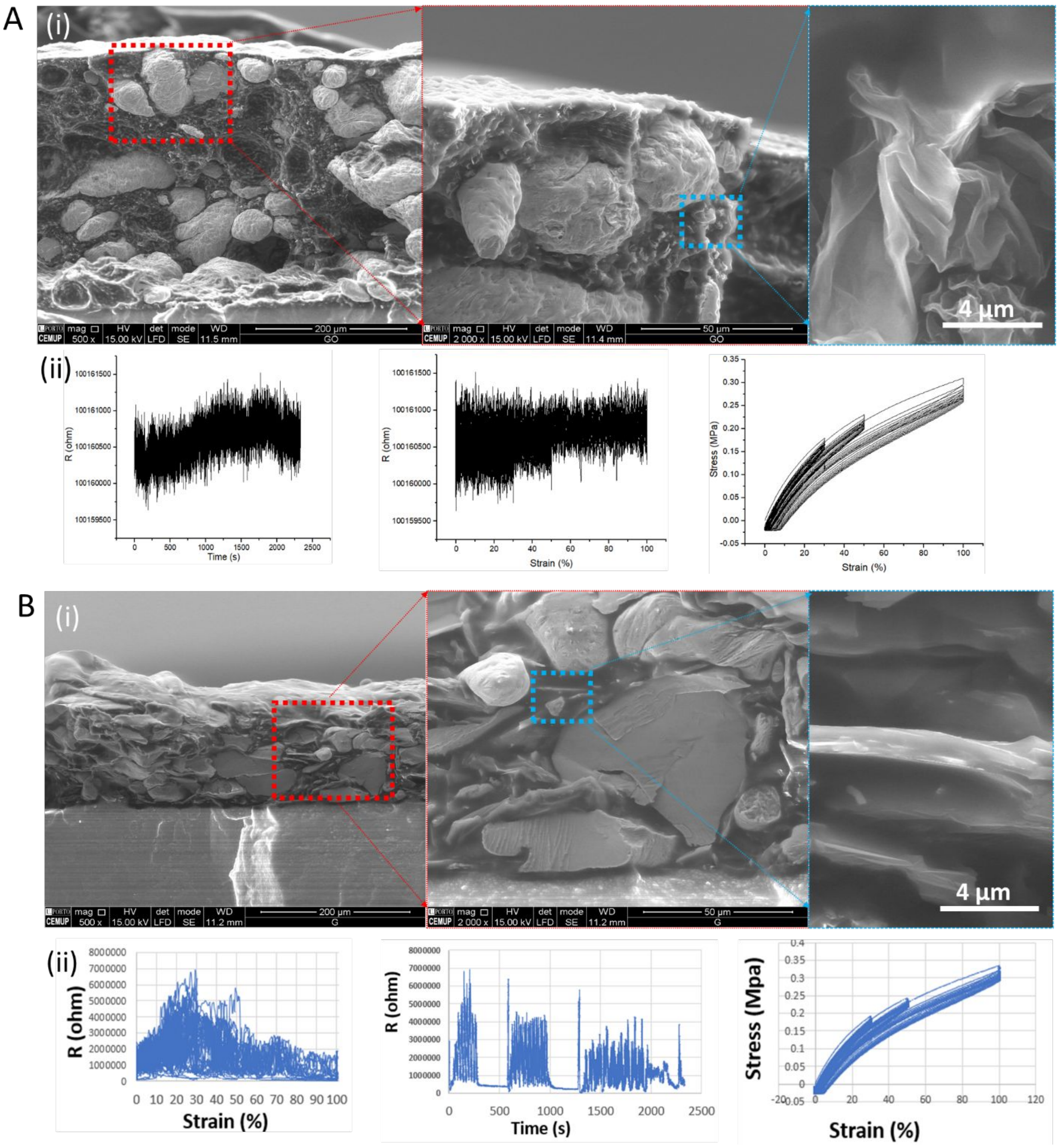
A
(i)
mag 500 x
HV 15.00 kV
det LFD
mode SE
WD 11.5 mm
200 µm
GO
mag 2 000 x
HV 15.00 kV
det LFD
mode SE
WD 11.4 mm
50 µm
GO
4 µm
(ii)
R (ohm)
Time (s)
R (ohm)
Strain (%)
Stress (MPa)
Strain (%)
B
(i)
mag 500 x
HV 15.00 kV
det LFD
mode SE
WD 11.2 mm
200 µm
G
mag 2 000 x
HV 15.00 kV
det LFD
mode SE
WD 11.2 mm
50 µm
G
4 µm
(ii)
R (ohm)
Strain (%)
R (ohm)
Time (s)
Stress (Mpa)
Strain (%)

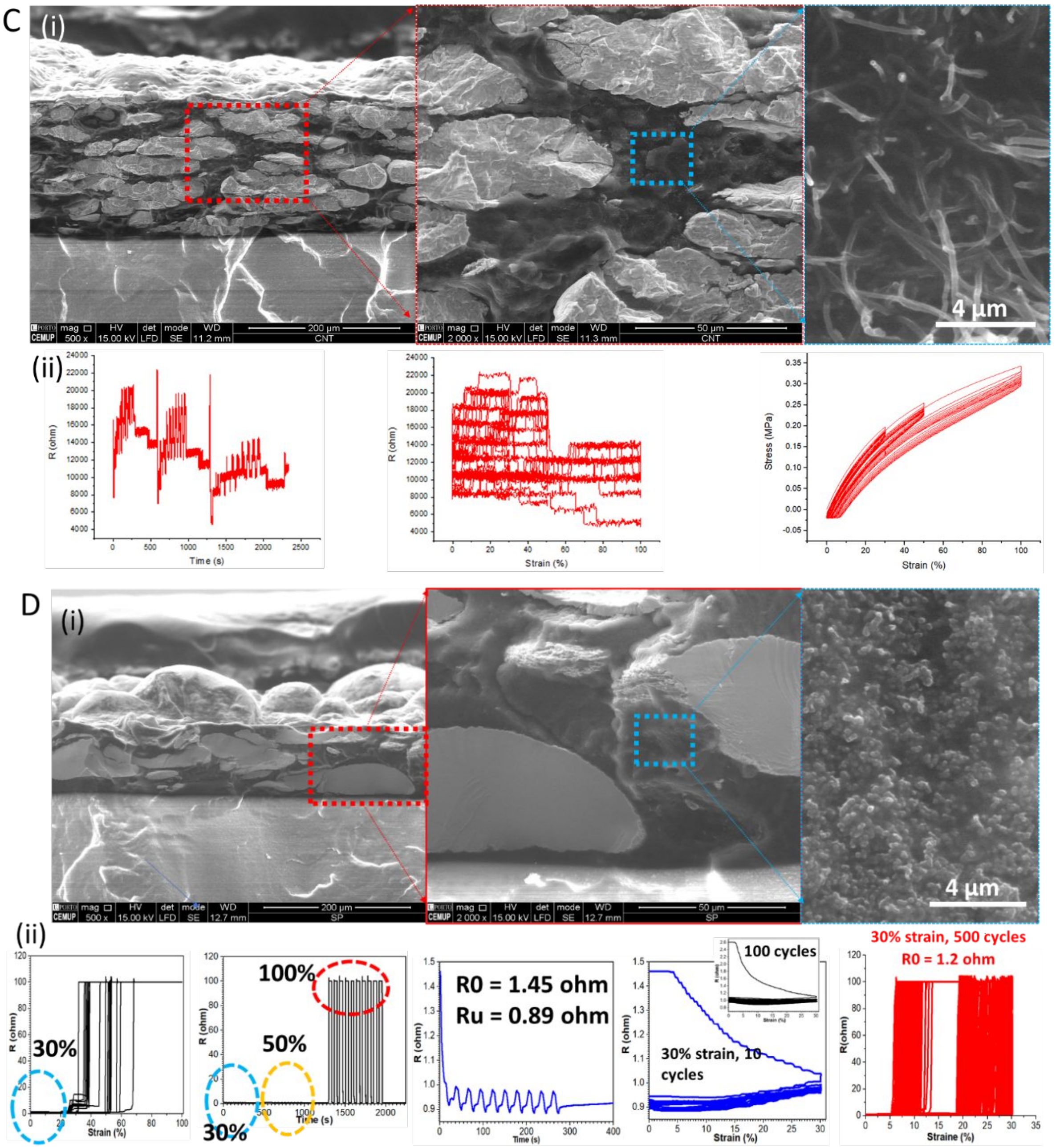

Figure S4. The C-Ga-SIS composite (CB: Ga weight ratio 0.043) with different types of carbon, **A)** Graphene oxide, **B)** Graphite, **C)** Multi-wall carbon nanotube, **D)** Super P conductive carbon. In all Figures, (i) Cross-sectional SEM-BS images and (ii) Electromechanical characterization under different strains (30%, 50%, and 100%) for 10 cycles and in the case of Super P conductive carbon under 30% strain for 100 and 500 cycles.

We initiated our study by blending CB, with SIS elastomer polymer to gain insights into the electrical and electromechanical (EM) behavior of the CB-SIS composite. The primary objective was to understand the impact of Ga on the CB-SIS composite, aiming to enhance its stretchability and achieve desired EM properties based on previous research that utilized filler particles such as Ag flakes, Ni, etc. [2, 15]. Subsequently, we introduced varying amounts of Ga into the CB-SIS composite and methodically investigated its EM behavior. The compelling results we obtained encouraged further exploration. Consequently, we tested this composition with other types of carbons, such as Graphene oxide (GO), Graphite (G), Multiwall carbon nanotube (MWCNT), Super P conductive carbon (SPCC), and Carbon black (CB), named as X, as their EM behaviours are illustrated in Figure S4. In this context, we synthesized the Ga-X-SIS composite (X: Ga weight ratio 4.3%). **Figure 2D** and **Figure S3** show the cross-sectional SEM-BS images of these composites. The cross-sectional images of the Ga-X-SIS composite are shown in **Figure 2D** and **Figure S3**. The images of the Ga-CB-SIS and Ga-SPCC-SIS composites are almost similar to each other, where carbon particles are distributed among the Ga droplets and SIS. This is while the images of the Ga-GO-SIS, Ga-Gr-SIS, and Ga-MWCNT-SIS composites indicate that the Ga droplets/particles are distributed among either sheets or tubes of the carbons. According to **Figure 2F** and **Figure S4**, the Ga-SPCC-SIS and Ga-CB-SIS composites presented better electrical and electromechanical properties. The Ga-CB-SIS composite presented higher stretchability compared to the Ga-SPCC-SIS composite. The Ga-SPCC-SIS composite could only withstand 30% strain for 10 cycles. But the Ga-CB-SIS composite could stand for 100% strain (**Figure 2F**).

Various reasons contributed to the better performance of CB compared to other composites. For instance GO inherent conductivity is lower than CB, and it requires a reduction reaction to form reduced graphene oxide to gain conductivity. Graphite on the other hand is a better conductor compared to GO. However, the size of graphite layers are too big compared to CB nano particles. Therefore, they are not able to play the role of high-suface area conductor that is desired within Ga-C-SIS composite. MWCNTs are well-known to be able to obtain high conductivity at low percolation threshold. Indeed we were expecting that MWCNTs present better electrical and electromechanical performance compared to CB. However, achieving homogeneous dispersion of

carbon nanotubes (CNTs) in elastomeric polymer nanocomposites poses a significant challenge. This dispersion is crucial for maximizing the enhancement of various composite properties [31-32]. Moreover, the low percolation threshold of MWCNT is due to their high aspect ratio shape, that promotes many conductive contacts, which does not seem to be a benefit in case of our composite. This is because in our composite, gallium has higher conductivity and dictates the final conductivity of the composite. And MWCNT are not able to provide the 3D structure to hold Ga, similar to the CB.

SPCC typically consists of small, spherical-shaped particles, providing a more uniform distribution within the composite. This facilitates the formation of continuous conductive paths, resulting in good electrical conductivity as observed in your study. Although SPCC overall properties were better than MWCNT, G, and GO, it did not present a significantly better property compared to the CB Therefore, it is not beneficial to use SPCC, which is as well more costly compared to the CB.

CB particles are highly conductive and have a spherical morphology, allowing for better dispersion within the composite. The spherical shape and efficient percolation pathways contribute to enhanced electromechanical behavior.

In summary, we can emphasize that the observed superiority of CB-Ga-SIS composite over other types of carbons in this study is likely due to its spherical morphology, better dispersion, and possibly enhanced interfacial interaction with the matrix, all of which contribute to more efficient percolation pathways and superior electromechanical behavior in the Ga-CB-SIS composite.

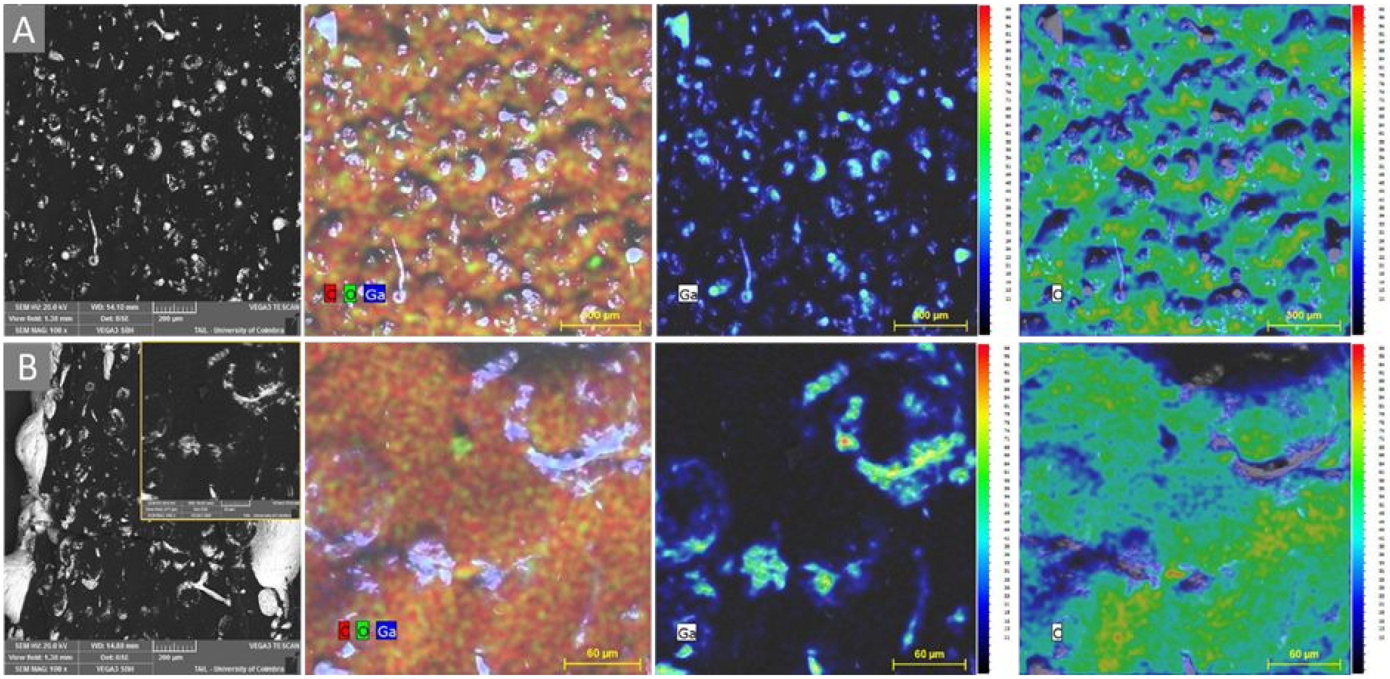

Figure S5. SEM-SE images and color map of the gallium and carbon black in the Ga-CB-SIS composite (CB: Ga weight ratio 0.043), **A)** pristine sample, **B)** after 10 cycles of strain-stress test.

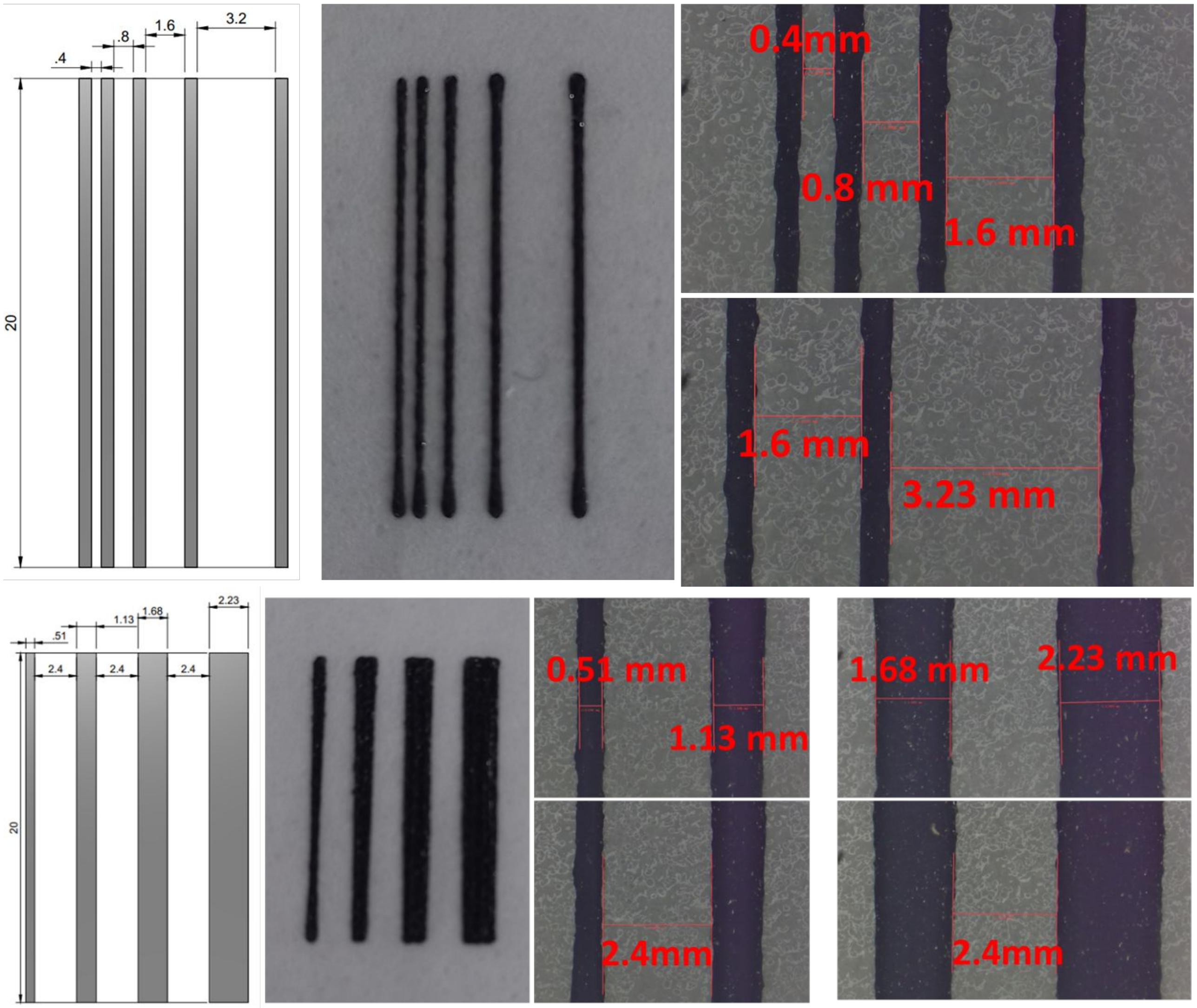
.4
.8
1.6
3.2
20
0.4mm
0.8 mm
1.6 mm
1.6 mm
3.23 mm
.51
1.13
1.68
2.23
2.4
2.4
2.4
20
0.51 mm
1.13 mm
1.68 mm
2.23 mm
2.4mm
2.4mm

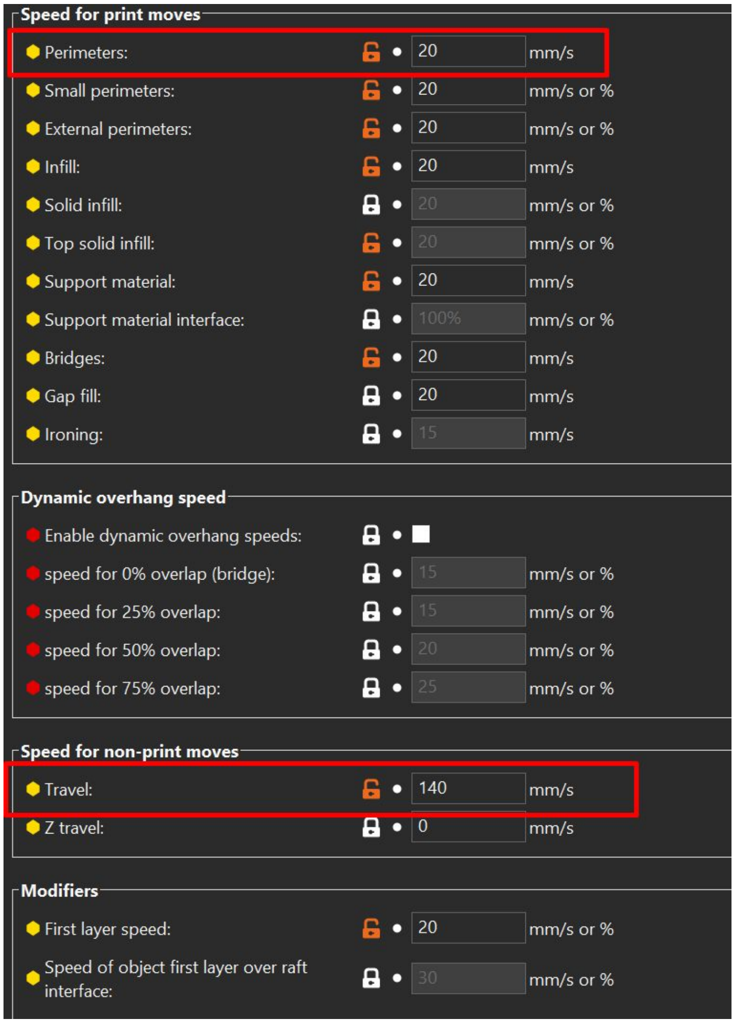

Figure S6. This indicates the printing states of a benchmark, such as lines with different widths and spacing with their corresponding resolution (based on the printer settings shown under the printed images).

With the proper printing preparation, we achieve high-resolution printing of circuits using nozzles of 200 or 250 µm. As shown in Figure S6, a minimum line spacing of 400 µm and a minimum linewidth of 510 µm could be obtained. The limitation visible in this scenario is not due to the materials per second. But to the fact that nozzles smaller than 200 µm, are usually very thin and bend easily, which during the machine calibration that requires contact between the nozzle and table, these needles tend to break. Indeed, over the past years, our research group has been focused on the development of a machine to print our new types of innovative inks. This type of printer allows us to leverage FDM (fused deposition manufacturing) technology to extrude inks in a controlled manner. We are now able to design a prototype circuit and transfer it to the material world using our proprietary digital fabrication method. In this article, we demonstrate the printability of the Ga-CB-SIS

composite with a gradient of linewidth and line spacing (Figure S6). For the machine settings, we printed both samples with a speed of 20 mm/s.

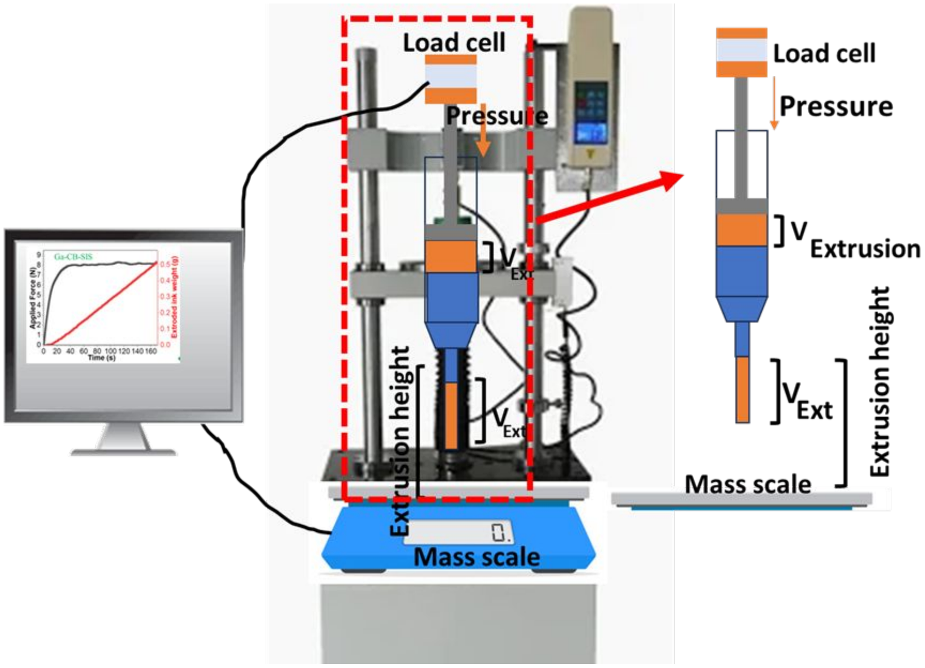

Figure S7. Schematics representing the setup used for extrusion testing.

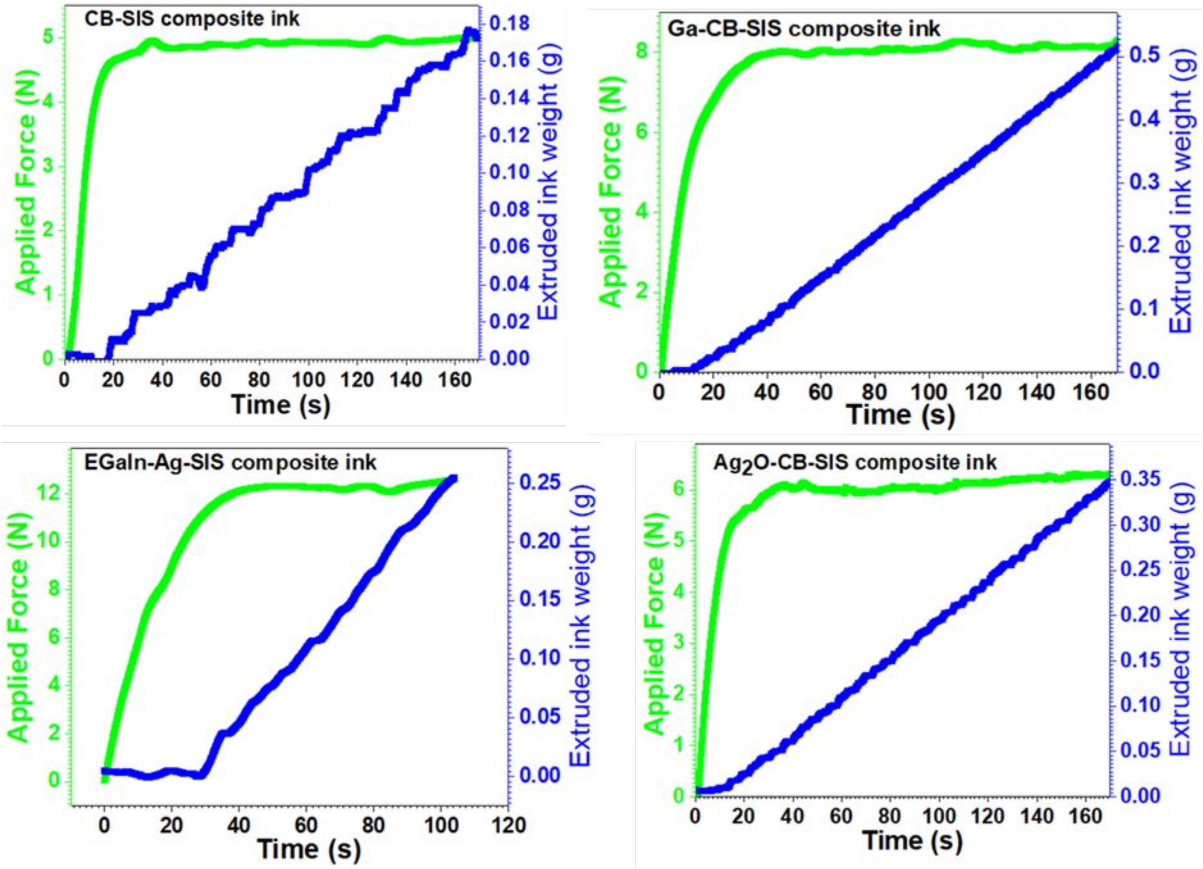

Figure S8. Characterization of the ink composites. Ag-EGaIn-SIS at 0.1 mm/min. Second current collector (CB-SIS) at 1 mm/min. Ag_2O-CB-SIS ink at 0.5 mm/min. Ga-CB-SIS at 0.5 mm/min.

Digital printing

In this research conducted in our laboratory, we developed specific printing profiles for each composite to ensure smooth printing, excellent resolution, and coverage when using a digital printer. Various parameters had to be adjusted for each ink to achieve smooth extrusion and adequate coverage without overprinting.

According to the digital printer settings (In a Voltera printer), the following parameters are defined:

- Rheological Setpoint: Adjusts the pressure gradually to compensate for viscosity differences in inks. Higher values result in a more gradual pressure adjustment, enabling homogeneous deposition along the trace length.

- Trim Length: Determines the length of the trace deposited with each pressure pulse. Adjusting this setting allows for enhanced ink deposition for small lines or longer lines.
- Feedrate: Specifies the speed at which ink is transferred to the substrate.
- Kick: Regulates the initial piston movement when pressure is applied at the start of each line, with higher viscosity fluids requiring a stronger kick.
- Soft Stop Ratio: Establishes the distance at which pressure stops being applied to the syringe plunger before reaching the end of the trace.
- Soft Start Ratio: Controls the delay between the kick and the start of printing.
- Pass Spacing: Controls the distance between parallel printed lines.
- Anti-Stringing Distance: Addresses issues of string formation by breaking strings formed during nozzle-to-board connections.

In this study, further investigation and adjustments to these parameters were made to achieve more homogeneous printing, prevent crack formation, and optimize material deposition. Specifically, reducing the pass spacing and trim length allowed for closer parallel lines and increased material deposition in a given area, leading to decreased crack formation.

We conducted multiple tests on various composites and optimized them based on the results obtained, utilizing the setup illustrated in Figure S7. In Figure S8, we present a characterization of different inks, including the EGaIn-Ag flake-SIS composite (serving as the first current collector in the fabricated battery), CB-SIS composite (acting as the second current collector), Ga-CB-SIS composite, Ag_2O-CB-SIS composite (used as the cathode in the battery), and Ga-CB-SIS composite (utilized as the anode in the battery). It's noteworthy that, for the Ag-EGaIn-SIS composite, a 0.5 mm nozzle was selected due to its capability to handle good flow and achieve uniform ink deposition, facilitating the printing of finer lines. Conversely, for inks containing carbon, a larger nozzle (0.7 mm) was employed to prevent clogging, given that the viscosity of the composite made it challenging for smaller nozzles to extrude the material.

In order to provide quantitative parameters for extrusion printing, we characterized the shear-thinning of ink composites. The procedure consisted of extruding each composite from the syringes employed for printing. An Instron mechanical testing device was used to apply a constant displacement rate while measuring the force applied to the syringe plunger. Simultaneously, a precise balance was placed at the bottom of the syringe to capture the moment of ink deposition. In this way, the abrupt drop in the applied force indicates the shear thinning force for each composite. The syringes had a diameter of 12.5 mm. For inks containing carbon, namely CB-SIS, Ag_2O-CB-SIS, and Ga-CB-SIS, a tip with a diameter of 0.4064 mm was utilized, while for Ag-EGaIn-SIS, a tip with a diameter of 0.245 mm was used. The results for all the composites are presented in Figure S8. Note that the extrusion rate of Ag-EGaIn-SIS is lower than the other composites, as due to the existence of LM, a lower extrusion rate is preferred for avoiding undesired extrusion of the ink from the syringe.

Rheological Properties

To assess the ink's rheological properties, extrusion tests were performed using a custom setup. This setup involved a tensile machine equipped with a load cell, connected to a mass scale positioned beneath it (see Figure S7). The same cartridges and nozzles utilized for printing were filled with ink, and the machine applied force to extrude the ink over a 2 mm distance. Force exertion ceased after reaching this point. The behavior of each ink was recorded, as shown in Figure S8, illustrating extrusion force and mass over time.

In the graphs, each ink exhibited a specific minimum force required to initiate extrusion. Prior to reaching this threshold, pressure built up until the ink started flowing. Once flow commenced, the force stabilized, and the ink extruded at a relatively consistent rate. Due to pressure accumulated in the cartridge, ink typically continued to pour out of the nozzle for a short period after force exertion ceased. During this phase, the force gradually decreased while the total mass of the extruded material, measured by the scale, increased.

Based on the extrusion tests, it was observed that the Ag_2O-CB (cathode) ink required the highest force for proper extrusion compared to other inks. This aligns with practical experience during the printing process, where Ag_2O ink proved particularly challenging. This higher force requirement can be attributed to the ink's high mass loading and large particle size (Ag_2O particles typically range from several micrometers to tens of micrometers), posing obstacles to achieving smooth ink flow and uniform deposition.

Additionally, in the conducted tests, the Ag-EGaIn-SIS (1st CC) ink utilized a nozzle with an internal diameter of 0.254 mm, while the other three inks (Anode, Cathode, and 2nd CC) employed a larger nozzle with an internal diameter of 0.40 mm. This disparity is due to the 1st CC ink being more "print-friendly" owing to its lower viscosity, greater ink uniformity resulting from smaller particle sizes, and inclusion of liquid metal. Consequently, the 1st CC ink can be uniformly extruded even with smaller syringe diameters, facilitating higher-resolution printing. Conversely, the other three inks necessitated a larger nozzle size due to their distinct characteristics. The applied shear rates calculated for the inks were 0.525 s^{-1} for the 1st CC and 0.333 s^{-1} for the other three composites, computed based on the formula $\gamma = 4Q/\pi R^3$, where R is the inner radius of the syringe, and Q is the volumetric flow given by $Q = \pi R^2 h/t$, with h representing the printing height and t the time.

A limitation of the test is the distance between the extrusion point and the mass scale, resulting in a delay between load cell measurement and scale reading. This distance is necessary to prevent ink accumulation caused by movement in axes other than the z-axis, which could disrupt precise measurement. Another observed issue is the occasional accumulation of ink on the tip of the nozzle, which subsequently drops down to the surface of the balance after a while. This can explain the delay in ink pouring and slight deviations in linearity during measurement.

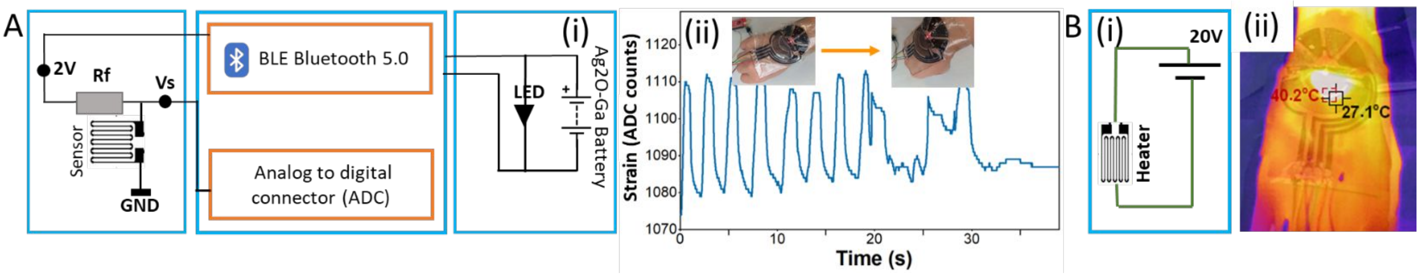

Figure S9. A) Schematics diagram exhibiting the working principle and main components of the Sensor−PCB (i), The recorded results via Bluetooth (Strain (ADC counts) vs. time (s) (ii), **B)** the working principle and main components of the Heater−PCB (i), Thermal image of heater (ii).

Table S1: Characteristics of the previous works compared to this work: Based on the composite, electromechanical properties, printability, and application.

Materials/composites	Max. strain	Conductivity/Resistance	Digitally Printable	Application	Ref.
PDMS-MWCNTs-EGaIn	120%	1.315× 10^{-2} $S.cm^{-1}$ to 7.02 × 10^{5} $S.m^{-1}$	No	Strain sensor, Capacitance sensor	[3]
CNT-LM-PDMS	50%	4.9 × 10^{-4} $S.cm^{-1}$	No	sensor	[4]
LM-X-SIS (X= Ag flakes, Ag-coated-Ni, Ag-coated-Fe, Ni, Ferrite, and TiC)	500%	7.02 × 10^{5} $S.m^{-1}$	Yes	Sensor	[2]
LM@PDMS	>100%	18.1 $S.m^{-1}$	No	Heater, 2.0 V for 50 °C	[5]
AgNW-LM-elastomer hybrid composite	90% strain	(3.41 ± 0.57) × 10^{5} $S.m^{-1}$	No	Heater, 5.5 V 110 °C	[6]
(CNTs)/carbon nanofibers (CNFs) and liquid metal/PDMS	>300% strain	33 $S.m^{-1}$	No	Heater, 5.5 V	[7]
AgNW/LM/elastomer	1000%	(1.31 ± 0.13) × 10^{6} to (7.36 ± 0.69) × 10^{5} $S.m^{-1}$	No	Sensor Heater, 5.5 V	[8]
Cu-EGaIn-PVA glue	75%	6 × 10^{6} $S.m^{-1}$	No	Heater, 58 °C	[9]
EGaIn-polymethacrylates (PMA) glue	20%	-0.4 $S.m^{-1}$	No	sensor	[10]
Ni-EGaIn	-	1.61 × 10^{6} $S.m^{-1}$	No	Sensor, heater 45 °C	[11]
LM-SIS/PBD	>700%	~10 $S.m^{-1}$	No	Heater 150 °C, sensor, actuator	[12]
LM-SBS	800%	12 000 $S.cm^{-1}$	No	sensor	[13]
EGaIn-Ecoflex-Ni	>200	<1× 10^{-6} $S·m^{-1}$	No	Heater, ~48 °C	[14]
LM-Ni-SIS	>500%	1.33 $S.m^{-1}$	Yes	Magnetic sensor-swicher	[15]
Ga-CB-SIS	>500%	0.25 $S.m^{-1}$	Yes	Heater,sensor,battery integrated	This work

Table S2: Characteristics of the previous works compared to this work: based on materials, electrical properties and strengths and weaknesses.

Materials	Category of Materials	Limit of Electrical Properties	Strengths and Weaknesses	Ref.
Metallic /nanoparticles/ flakes /nanowires (e.g., Cu/Ag/AgNWs/Au/Ni/Al)	Metal and its derivatives	$\approx 10^4$–6.3×10^7 $S.m^{-1}$	Extremely conductive<br>Resistant against air ageing<br>Inflexible<br>Less comfort	[16]
CB/CNF/ SWCNT/MWCNT /GO/rGO/MXene/	Carbonaceous materials	$\approx 10^2$–10^9 $S.m^{-1}$	Highly conductive and stretchable<br>Time-consuming process	[17-18]
PANI/PPy/PEDOT: PSS/PhT	Intrinsically conducting polymers	≈ 10–1.7×10^{-3} $S.m^{-1}$	Less cost and density<br>Non-resistant to air ageing	[19]
LM-CB-SIS	Carbonaceous materials+LM	≈ 0.25 $S.m^{-1}$	Highly Conductive<br>Stretchable<br>Digitally printable<br>Less Cost<br>Easy process	This work
